# Lightweight Anonymous Authentication and Key Agreement Protocol Based on CoAP of Internet of Things

**DOI:** 10.3390/s22197191

**Published:** 2022-09-22

**Authors:** Xiang Gong, Tao Feng

**Affiliations:** School of Computer and Communication, Lanzhou University of Technology, Lanzhou 730050, China

**Keywords:** CoAP, Internet of Things, authentication, anonymity, key agreement, CPN Tools

## Abstract

To solve the problem regarding the lack of a lightweight and secure authentication and key agreement protocol in the Constrained Application Protocol of the Internet of Things environment, we explore the security flaws and applicability problems in the current related research. Then, we propose a new lightweight authentication and key agreement protocol based on the CoAP framework. The scheme adopts shared secret and elliptic curve public key technology, which ensures the anonymity of the communicators and provides strong security and anti-attack capacity. In terms of security analysis, the Dolev–Yao Adversary model and a security model checking analysis method based on CPN Tools are improved, in order to verify the correctness and security of the proposed scheme. Compared with other schemes, regarding communication overhead, computational cost, and security, the proposed scheme provides a robust and comprehensive security guarantee, although it is not the lightest.

## 1. Introduction

Constrained Application Protocol (CoAP) is a lightweight communication protocol developed by the Internet Engineering Task Force (IETF) for the Restricted environment of the Internet of Things (IoT) [1]. CoAP has gradually become the most popular protocol in IoT applications, due to its ease of implementation, low power consumption, lightweight communication, high mobility, good compatibility with Hyper Text Transfer Protocol (HTTP), and sufficient technical space to enhance data security and integrity [2]. Motaharul et al., have proposed a cloud-based IoT architecture and coordinated IoT using the CoAP protocol [3]. Their evaluation experiment showed that the CoAP protocol was more suitable than Message Queuing Telemetry Transport (MQTT) to realize communication between sensors in the cloud computing environment. In addition, CoAP has been quickly adopted and supported by many large companies. Researchers have introduced it in many fields, from smart homes to industrial wireless sensor networks [4].

**Figure 1:**
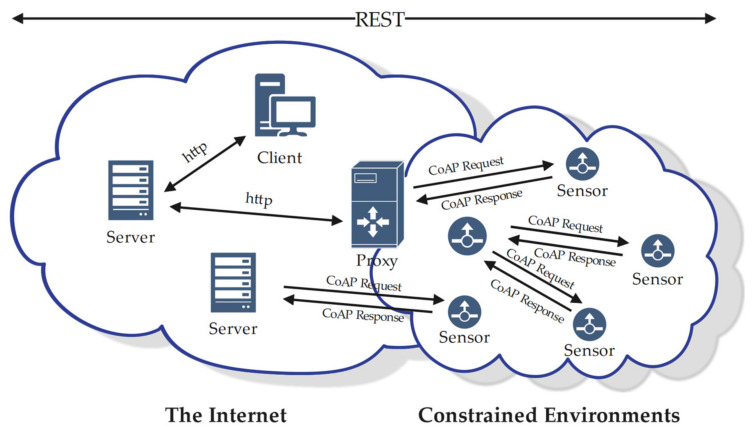
Network framework of CoAP.

CoAP is a client server-based protocol, representing a lightweight implementation of HTTP protocol based on the REST architecture (see Figure 1). In order to overcome the disadvantages of HTTP for restricted environments, CoAP takes into account both the optimization of datagram length and reliable communication. On one hand, CoAP provides scalability by providing URIs, restful methods (e.g., GET, POST, PUT, and DELETE), and header options that can be defined independently; on the other hand, it is based on the User Datagram Protocol (UDP) protocol and allows IP multicasting. CoAP defines a transaction processing mechanism with a re-transmission mechanism, in order to compensate for the unreliability of UDP transport. Moreover, it provides a resource discovery mechanism with resource description.

CoAP does not blindly compress the HTTP protocol. Considering the low processing power and low power limitations of resource-constrained devices, CoAP re-designs some HTTP functions, adapting them to the constrained environment. In addition, to adapt the protocol to IoT Machine-to-Machine (M2M) applications, the CoAP protocol improves some mechanisms and adds some functions. These include piggybacking messages and Observe patterns.

As there is no reliable security system standard, the security of CoAP protocol has always been an important topic. CoAP uses UDP as the transport layer protocol. In the initial design of CoAP, the designer adopted Datagram Transport Layer Security (DTLS, RFC 6347) as the security protocol [1]. However, DTLS was not initially designed for resource-constrained IoT environments; for example, to perform a handshake, DTLS must transmit messages up to six times, adding communication overhead and consuming more power on the device.

Furthermore, attacks against UDP or DTLS can also affect CoAP. Attacks on DTLS can be launched in a single session and require enhanced authentication mechanisms. Man-in-the-middle (MitM) attacks are one of the most severe security problems in CoAP, as cited in RFC 7252 [1]. These include sniffing, spoofing, denial of service (DoS), hijacking, cross-protocol attacks, replay attacks, and so on. Various researchers have studied DTLS, in order to determine how it protects CoAP communication messages.

In recent years, the IETF has been committed to the improvement and security protection of the CoAP protocol. To date, it has issued several RFC documents that complement and improve CoAP. Specifically, RFC8613 [5] specifies a security protocol called Object Security for Constrained RESTful Environments (OSCORE) for the environment with CoAP, which is a method of application-layer protection for CoAP encoded using Concise Binary Object Representation (CBOR, RFC8949) [6] and signed and encrypted using Object Signing and Encryption (COSE, RFC8152) [7]. Compared with DTLS, OSCORE adopts lightweight processing and effectively guarantees security. It also eliminates the problem of malicious agents using DTLS [5], providing an effective security guarantee for CoAP.

However, the OSCORE protocol does not include a key agreement phase, which assumes that the materials required for the session key have been exchanged between the communicating parties before running. Therefore, excluding the inapplicable DTLS protocol, the CoAP secure key agreement protocol remains an open problem. Many researchers have proposed key agreement protocols for CoAP or similar IoT scenarios but, to date, there are still usability or security problems in these protocols. The aforementioned unique functions provided by CoAP, such as multicast and observe mode, all require reliable and secure session guarantees. Therefore, designers should design secure session key agreement protocols based on the unique properties of CoAP. In this paper, we describe a key agreement protocol based on CoAP, then conduct relevant security model checking and analysis based on CPN Tools.

The contributions of this paper include the following aspects:

We investigate the existing related solutions and their security and usability problems.

- We propose an anonymous mutual authentication key agreement protocol, which can satisfy the network architecture of CoAP and provide a robust security guarantee.
- We improve the Dolev–Yao Adversary model, and CPN Tools is used to describe and analyze it thoroughly.
- We demonstrate a security protocol modeling and security verification method based on CPN Tools.
- A comprehensive formal and non-formal analysis of the proposed protocol security is carried out.

The remainder of this paper is organized as follows: In Section 2, the relevant literature is reviewed. Our proposed protocol is described in Section 3. Section 4 formally verifies the model of the proposed protocol with CPN Tools. Section 5 analyzes the security of the protocol. The performance and security of the proposed protocol are compared with those of existing protocols in Section 6. Finally, our conclusions, the existing problems, and future work are discussed in Section 7.

## 2. Related Works

This section reviews related works and contains three parts: Secure Key Exchange Schemes for CoAP and IoT, Applicability of Existing CoAP Secure Key Exchange Schemes, and Formal Analysis Tools for Security Protocols.

### 2.1. Secure Key Exchange Schemes for CoAP and IoT

Initially, Villaverde et al. [8] proposed associating DTLS and CoAP to protect the security of CoAP. In 2014, this program was recommended by ITEF in RFC7252 [1]. However, as the new design of DTLS requires a header compression scheme, the end-to-end security attributes provided by the original DTLS protocol may be compromised. Its handshake scheme requires too much message fragmentation when adaptive, which means that the number of re-transmissions and re-ordering of DTLS handshake messages is too high, making it unsuitable for resource-constrained devices [9]. In addition, although CoAP supports multicast connections, DTLS only protects unicast messages [10,11,12,13].

Arvind et al. [14] have introduced a series of attacks against CoAP and DTLS, such as man-in-the-middle attacks, sniffing attacks, spoofing attacks, DoS attacks, hijacking attacks, cross-protocol attacks, replay attacks, and so on. Their research showed that the security of CoAP needs to be further improved in practice, and there should be more targeted lightweight security schemes available to ensure the security of CoAP.

Figueroa et al. [15] have proposed a security protocol named LSPWSN. Web services often cannot be easily used under limited resource scenarios, such as in sensor nodes. LSPWSN aims to overcome this problem and provide secure web services with limited resources. It follows a RESTful approach and encodes headers and payloads using binary encoding. The authors simulated LSPWSN and CoAP on the Contiki Cooja network simulator, and showed that LSPWSN consumes nearly five times more resources than CoAP, with nearly four times more net packets than CoAP. Therefore, LSPWSN may not be applicable, due to performance degradation. Further improvements are needed to improve the performance of LSPWSN.

Van et al. [16] have proposed a reverse proxy approach to overcome RESTful end-to-end security and performance problems in constrained environments. The authors believed that the identified problems could be solved using the reverse proxy approach, which splits end-to-end security on the proxy. Therefore, the Security Service Proxy (SSP) was proposed to provide additional functions and services on constrained networks and nodes. The primary goal of SSP is to reduce overhead and improve the performance and functionality of constrained RESTful environments. SSP has achieved good results, but it also has some limitations. It introduces a single point of failure, in terms of security and operation. In addition, SSP can quickly lose all sessions and public/private keys, in the case of a compromise [16].

Ukil et al. [17] have proposed a CoAP lightweight security protocol for IoT applications, based on the Advanced Encryption Standard (AES) algorithm and with key length of 128. It is fast, efficient, adaptable to different IoT applications, resilient to replay and man-in-the-middle attacks, and has low overhead; however, this protocol suffers from the defects described in [4]. The authors assumed that pre-shared keys are hard-coded into each device at the time of manufacturing, which is not always applicable. It is recommended that the program be improved to work without such assumptions, in order to assess the proposed technical performance.

Bhattacharyya et al. [18] have proposed a lightweight key agreement protocol, named LESS, for the session key agreement process of CoAP. After the session is established, control is switched from CoAP to DTLS to provide channel security; however, the protocol does not provide anonymity, forward secrecy, and is vulnerable to Key Compromise Impersonation (KCI) attacks.

The solution proposed by Nathi et al. [19] aims to achieve lightweight identity authentication and security protection on the CoAP, where the payload is encrypted based on the mutual service protocol between the client and the server. The data exchange phase is initiated with the server application, which does not contain any handshake mechanism and does not have a key exchange algorithm. The security scheme uses two rounds of encryption and decryption to ensure the security of communication on the network. However, in the network deployment process, an applicability problem appears, as described in the work [4]. The shared key needs to be written into the device’s read-only memory (ROM) when leaving the factory, which limits the scalability of the network.

Suman et al. [20] have explained the shortcomings of the LESS protocol [18] in their work and named their protocol ECC-COAP, claiming that it improved the LESS protocol and reduced the number of flights to five. They used the AVISPA tool to verify the protocol, and their results indicated that it can provide adequate security. However, the protocol does not provide anonymity, the network overhead of the protocol is too significant, and the protocol does not provide forward and backward secrecy under the assumption that the adversary obtains the private key of both parties.

Abosata et al. [21] have proposed a lightweight authentication optimization protocol for distributed sensors based on payload encryption, which integrates and optimizes the authentication mechanism of DTLS in the CoAP architecture. However, the alias ID and the shared secret used in the protocol are fixed, such that there is no unlinkable attribute. Therefore, the system will have great hidden trouble when the pre-shared information is compromised. In addition, the server needs to store all the expired one-time random nonces, which requires a significant storage expense in the network with numerous nodes and long-term existence.

Oliver et al. [22] have proposed a lightweight CoAP mutual authentication protocol using the Shared secret and XOR, hash calculation, symmetric encryption, and AES back-and-forth in a message for the entire process; in terms of computational load and network load, this protocol provides extremely lightweight services. However, the scheme security is only provided by a shared secret key. The session key combines plain-text random numbers, which do not provide anonymity and forward secrecy, such that there are significant security risks when the key is compromised.

In a word, the existing key exchange schemes of CoAP still have many defects, and researchers need to invest more in their analysis and design. However, secure key exchange schemes are also essential to the entire IoT. Recently, various security protocols have been proposed. We studied two that utilize the ECC algorithm and have similar application scenarios as the proposed protocol. One is a device control and key agreement protocol for IoT called LACKA-IoT, proposed by Das et al. [23]. The other is a two-party authentication protocol for IoT, proposed by Alzahrani et al. [24]. It can be stated that these two works have security flaws [25]. We will compare our protocol with these works [20,21,22] with regard to performance and security in Section 6.

### 2.2. Applicability of Existing CoAP Secure Key Exchange Schemes

As CoAP is an M2M protocol, it has been stipulated, in RFC7252, that the client initiates the direction of resource discovery and observe mode to the server. However, in reality, the server initiates a request to the sensor for data (as shown in Figure 1). As a result, researchers have potential conflicts in their understanding of CoAP. In the Internet of Things employing CoAP, it is necessary to explain who play the roles of client and server.

The network architecture of CoAP differs from the usual Server–Client architecture, in that the sensor is thought of as the client, that is, the party that holds the resource. The device that initiates the request is usually a gateway or server in the network with no resource limitations. This provides the possibility of device discovery, the observe mode, piggybacks, and multicasting. As can be seen from the CoAP framework, the device requests data from the sensor, and the sensor is the data holder responsible for responding to and providing data to the Initiator, that is, as an M2M communication protocol, CoAP allows nodes to play the role of both Initiator and Responder. However, sensors with limited resources as end nodes should only respond without providing the function of initiating data, as they require data from users and taking into account their limited hardware. This has been previously explained (see, e.g., [1,18,26]). However, the CoAP network architecture assumed in the literature [22] is the same as that in [20]. These contrary request–response assumptions will lead to implementation contradictions, where either the lightweight or discovery and observation characteristics of CoAP will be abandoned.

Therefore, the scenario proposed in this article assumes that an Initiator with unlimited resources initiates the request. The Initiator and Responder are one-to-many or few-to-many. An Initiator stores the pre-shared information of all Responders, while a restricted Responder (such as a sensor) stores the pre-shared information of one or more of the Initiators. To avoid ambiguity, “Initiator” and “Responder” are used instead of “client” and “server”.

### 2.3. Formal Analysis Tools for Security Protocols

The model checking procedure generally consists of four basic steps. The researcher thoroughly studies the protocol standard/specification in the first step. The second step involves formal modeling, according to the given specification. In the third step, a model checker performs an automatic formal verification. In the last step, the formal verification results are output, and the standard/specification is upgraded, based on these results [27].

The model check software CPN Tools used in this paper is a Colored Petri Net (CPN) tool developed by Aarhus University of Denmark, a visual CPN simulation analysis tool. The model can be run directly on the UI interface when the modeling is completed. It enables researchers to intuitively observe the various states and performances of the model at run-time. It implements the modeling and simulation of high-level Petri nets and introduces SML/NJ as an auxiliary language. The tool provides the ability to conduct state space analysis. It can automatically calculate the state space and generate reports [28]. After the state space is computed, a state space search can be performed through customized coding, making formal model checking possible [29]. In addition, this tool also provides functions such as monitoring and time attribute extension [30].

Compared with other formal verification tools for security protocols, such as ProVerif, Scyther [31], AVISPA, and so on, CPN Tools possesses unique advantages. In [32], CPN Tools was compared with various other automated verification tools. The authors claimed that the main advantage of CPN Tools was in the extraction of attack traces of the protocol. When the possible vulnerabilities of the protocol are found, the attack traces extracted by ProVerif and AVISPA were relatively single and, so, they cannot fully determine the possible flaws of the protocol. Scyther’s Adversary model was considered the most potent Adversary model in [30], which can extract multiple attack traces. However, compared with ProVerif, AVISPA, and other cryptographic verification tools, it is limited by a fixed algorithm and lacks support for newer cryptographic primitives (e.g., homomorphic encryption, attribute encryption, and so on), which are defined in the analysis of some security protocols. At the same time, the algorithm also limits Scyther’s search for protocol flaws. In an analysis of the TMN protocol [29], CPN Tools was found to be able to find security vulnerabilities that Scyther could not.

CPN Tools can be considered semi-automatic, compared to the above tools. It requires the modeler to input the description of the Adversary model and analyze each protocol uniquely, but this is also an advantage of CPN Tools. The high flexibility allows modelers to conduct targeted verification according to different security protocols, customize symbols and functions, describe cryptographic primitives according to their characteristics, and simulate various cryptographic primitive algorithms. New and more complex cryptographic primitives can still be emulated. After detecting protocol security problems, the number of attack paths extracted is based on the grade of the fine-grained model built, which makes the protocol vulnerabilities that CPN Tools can discover more accurate and comprehensive than other tools.

Furthermore, CPN Tools simulation can also analyze part of the protocol’s performance. CPN Tools can simulate the network environment and concurrent running process based on suitable modeling. The correctness and robustness of the model can be experimented with by setting the running times and setting appropriate observation places or monitors [32,33].

In recent years, as the superiority of CPN security formal analysis has been realized, more and more researchers have paid attention to and utilized CPN Tools for protocol security analysis. Permpoontanalarp et al., have used CPN Tools to analyze the TMN protocol [34] and compared several protocol analysis tools. The authors proposed a method to apply an on-the-fly method to CPN Tools and successfully analyzed the security of the TMN protocol, finally providing an improved scheme. Igorevich et al., have analyzed security in the domain of an in-vehicle communication security protocol using CPN Tools [35]. Two known replay attacks on the protocol were modeled and demonstrated. Amoah et al., have used CPN Tools to analyze the DNP3-SA protocol [36]. They claimed to have found a previously undiscovered attack trace and provided an improved scheme. The improved protocol was verified by the CPN model, which proved the effectiveness of the CPN model and the importance of formal protocol analysis. Rodriguez et al., have used CPN Tools to model and analyze the MQTT protocol [32]. When verifying the protocol model, the authors used incremental model checking to reduce the impact of the state explosion problem. In this work, the quality of service (QoS) mechanism of MQTT was modeled, and the issues of different MQTT QoS were analyzed. Luo et al., have used CPN Tools to analyze the security of the Wireless Hart protocol [28], verified the known protocol vulnerabilities through state space analysis, updated the protocol for security flaws, and used CPN Tools to verify the updates again. In addition, other similar works [37,38,39] have used CPN Tools to conduct formal security analyses for the SIP protocol, 5G AKA and QKD protocol. From the above, it can be seen that CPN Tools play an essential role in the formal analysis of security protocols.

## 3. Proposed Scheme

The proposed protocol includes anonymity, mutual authentication, and key agreement. Considering perfect forward and backward secrecy and other security attributes, the scheme adopts technology based on the elliptic curve to make the protocol as lightweight as possible. The symbols used in the protocol are shown in Table 1.

The scheme consists of the Initialization and Registration phase and the Identity Authentication and Key Agreement phase.

### 3.1. Initialization and Registration

**Figure 2:**
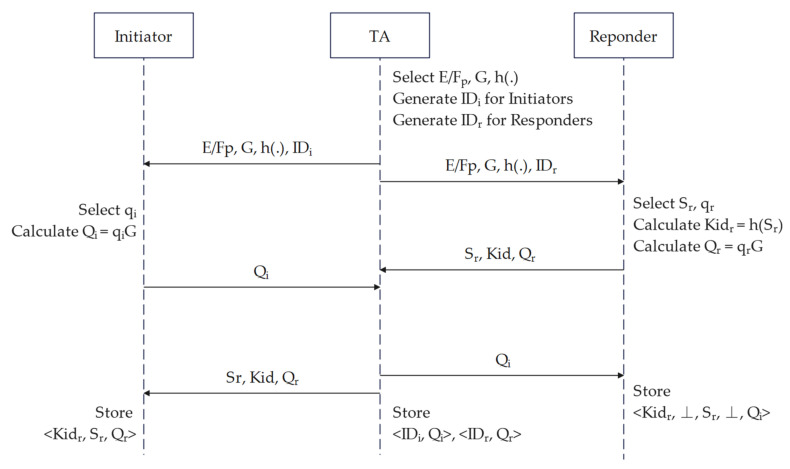
Initialization and registration phase.

In the first phase, the TA is assumed to exchange secrets with the Initiator and Responder through a secure channel. The role of the TA is to assign IDs to all devices and assist the devices in identifying each other initially and pre-sharing the secret. In addition, since the protocol is entirely anonymous, a TA is required to store all devices’ real IDs and public keys to achieve non-repudiation when necessary. The procedure of this phase is shown in Figure 2.

First, the TA selects an elliptic curve ${E/F}_{p}$, a generator G, and a one-way hash function h(.) over a finite field $F_{p}$ with sizeable prime order p (SHA3-256 is recommended). Except for the split (v1–v4) data, the first 128 bits of the hash string are truncated. Then, a 64-bit Extended Unique Identifier (EUI64) is selected for each device, as the device ID, and the essential data are sent to each registering device. The device will perform the calculation using the specified algorithm and save its ID.

Next, the Responder device randomly selects its first secret $S_{r}$, calculates the hash value ${\mathrm{Kid}}_{r}=h\left(S_{r}\right)$, selects the private key $q_{r}\in Z_{p}^{*}$ and stores it in secure memory, calculates the public key $Q_{r}{=q}_{r}G$, and sends the public key, $S_{r}$ and ${\mathrm{Kid}}_{r}$ to the TA. The Initiator selects the private key $q_{i}\in Z_{p}^{*}$ and stores it in secure memory. After calculating the public key $Q_{i}{=q}_{i}G$, it sends the public key to the TA. TA records the IDs and public keys of both parties. Moreover, the TA sends $S_{r}$, ${\mathrm{Kid}}_{r}$, and $Q_{r}$ to the Initiator, and $Q_{i}$ to the Responder. Upon receipt, the Responder will create and save the pre-shared data in its Non-Volatile Memory format ${<\mathrm{Kid}}_{r}{,\;\mathrm{pKid}}_{r}{,\;S}_{r}{,\;\mathrm{pS}}_{r}{,\;Q}_{i}>$ and initial values ${<\mathrm{Kid}}_{r},\;⊥{,\;S}_{r},\;⊥{,\;Q}_{i}>$. The Initiator creates and saves data to the database in the format and initial value ${<\mathrm{Kid}}_{r}{,\;S}_{r}{,\;Q}_{r}>$.

The Initiator also needs to save each Responder’s IP address, port, and other corresponding information in reality; however, this is irrelevant to the discussion in this paper, so it is not further discussed.

### 3.2. Identity Authentication and Key Agreement

**Figure 3:**
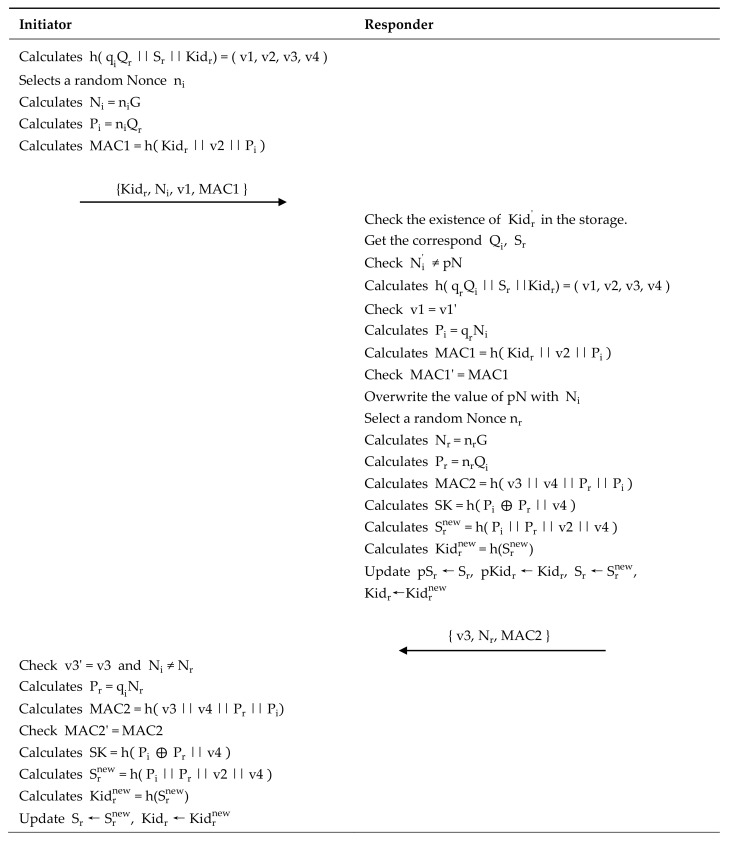
Identity authentication and key agreement phase.

This phase allows the Initiator and Responder to identify and trust each other, establish symmetric encryption keys required for subsequent data interaction, and update shared secrets. It consists of four steps (see Figure 3):

- Step 0: Responders power on

In this phase, the pre-shared data are read into memory, the static memory space is allocated for the long-term variable pN required by the corresponding communication object, and the error counter is set to an initial value of 0.

- Step 1: Initiator → Responder: $\left\{{\mathrm{Kid}}_{r}{,N}_{i},v1,\mathrm{MAC}1\right\}$

The Initiator finds the public key $Q_{r}$, ${\mathrm{Kid}}_{r}$, and the secret $S_{r}$ of the target, calculates ${h(\;q}_{i}Q_{r}{||S}_{r}{||\mathrm{Kid}}_{r})$ and divides the result evenly into $\left(v1,v2,v3,v4\right)$. Select a random nonce $n_{i}$, calculate $N_{i}{=n}_{i}G$ and $P_{i}{=n}_{i}Q_{r}$, calculate $\mathrm{MAC}1=h\left({\mathrm{Kid}}_{r}{||v2||P}_{i}\right)$, and send Message1 = $\left\{{\mathrm{Kid}}_{r}{,R}_{i},v1,\mathrm{MAC}1\right\}$ to the Responder.

- Step 2: Responder → Initiator: $\left\{{v3,\;N}_{r},\;\mathrm{MAC}2\right\}$

After receiving the request from the Initiator, the Responder uses the received ${\mathrm{Kid}}_{r}$’ to find the corresponding value in the storage. As the protocol stipulates that the latest and previous ${\mathrm{Kid}}_{r}$ values can be accepted, it first determines whether the ${\mathrm{Kid}}_{r}$’ value exists in storage. If it does not exist, the protocol is aborted, the error counter is incremented by 1, and the error is logged. If it exists, it takes out the corresponding Sr, Qi, and uses ${\mathrm{Kid}}_{r}$’ as the ${\mathrm{Kid}}_{r}$ value used in this process. The Responder checks whether the received value of $N_{i}$’ is the same as the previous value of $N_{i}$ (pN) stored by the Initiator. The received message is directly discarded without processing if the value is the same. If it is different, calculate ${h(q}_{r}Q_{i}{\;||\;S}_{r\;}{||\;\mathrm{Kid}}_{r})=(v1,\;v2,\;v3,\;v4)$, and check that the received v1’ value and the calculated v1 value are equal. If not equal, the protocol is aborted, the error should be logged, and the error counter increases 1. If equal, calculate $P_{i}{=q}_{r}N_{i}$, $\mathrm{MAC}1=h\left({\mathrm{Kid}}_{r}{||v2||P}_{i}\right)$, check the received MAC1’ value, and calculate that the MAC1 and MAC1’ values are equal. If not equal, the protocol is interrupted, the error is recorded, and the error counter increases 1. If equal, replace with the received $N_{i}$’ record of pN values corresponding to the Initiator. Choose a random number ${\;n}_{r}$, calculate $N_{r\;}{=n}_{r}G$, $P_{r}{=n}_{r}Q_{i}$, and $\mathrm{MAC}2=h\left({v3\;||\;v4\;||\;P}_{r}{\;||\;P}_{i}\right)$. Calculate the SK value and $S_{r}^{\mathrm{new}}$ and ${\mathrm{Kid}}_{r}^{\mathrm{new}}$ as follows: $\mathrm{SK}=h\left(P_{i}\;⨁\;P_{r}\;||\;v4\right)$, $S_{r}^{\mathrm{new}}{=h(P}_{i}{\;||\;P}_{r}\;||\;v2\;||\;v4)$, ${\mathrm{Kid}}_{r}^{\mathrm{new}\;}{=h(S}_{r}^{\mathrm{new}})$. Update the shared secret in the store: Replace ${\mathrm{pS}}_{r}$ with $S_{r}$, ${\mathrm{pKid}}_{r}$ with ${\mathrm{Kid}}_{r}$, $S_{r}$ with $S_{r}^{\mathrm{new}}$, and ${\mathrm{Kid}}_{r}$ with ${\mathrm{Kid}}_{r}^{\mathrm{new}}$. Finally, empty the temporary memory data and send response Message 2 = ${\{v3,\;N}_{r},\;\mathrm{MAC}2\}$ to the Initiator.

- Step 3: Initiator confirmation

The Initiator receives the response message from the Responder and checks whether the received v3’ is equal to the v3 value calculated in the previous step and whether the received $N_{r}^{\prime }$ value is not equal to the $N_{i}$ value calculated in the previous step. If there are any false results, the process is aborted and the temporary data in memory is cleared. If all is true, then calculate $P_{r}{=q}_{i}N_{r}$, $\mathrm{MAC}2=h\left({v3\;||\;v4\;||\;P}_{r}{\;||\;P}_{i}\right)$. Then, check whether the received MAC2’ value is equal to the calculated MAC2 value. If not, the Initiator does nothing and clears the temporary data from memory. If equal, then calculate $\mathrm{SK}=h\left(P_{i}\;⨁\;P_{r}\;||\;v4\right)$, $S_{r}^{\mathrm{new}}{=h(P}_{i}{\;||\;P}_{r}\;||\;v2\;||\;v4)$, ${\mathrm{Kid}}_{r}^{\mathrm{new}}{=h(S}_{r}^{\mathrm{new}})$. Finally, update the values corresponding to the database, replacing $S_{r}$ with $S_{r}^{\mathrm{new}}$ and ${\mathrm{Kid}}_{r}$ with ${\mathrm{Kid}}_{r}^{\mathrm{new}}$.

### 3.3. Subsequent Instructions

After the above protocol has run, both parties obtain the session key SK and the session is established. During the specified session period, the two communicating parties encrypt the message with the session key. CoAP can be encrypted with a symmetric encryption cipher suite, such as AES-GCM.

The error counter set in the Responder can reflect the situation of the device being attacked, to a certain extent. Therefore, once the counter value changes, the Responder can send an alarm message to the Initiator (including the protocol step), in order to find the error. This allows managers or proactive defense systems to identify the attack on the Responder, determine which part of the system is vulnerable, and decide whether to intervene.

As the transmitted messages do not include time stamps, the protocol does not require time synchronization between devices. However, in reality, the Responder should set several timers, according to the specific situation. For example, when the value of the error counter increases more than a certain threshold within a certain period, the Responder may send an alarm message to the Initiator and block the channel for a period to avoid DoS or battery depletion attacks. The Initiator can set the response window of the request message, and the request must be received within the specified time. Otherwise, the request is regarded as lost.

## 4. Formal Modeling and Security Verification

### 4.1. Adversary Model

The Dolev–Yao (DY) Adversary model has been widely used in the formal analysis of security protocols. The traditional DY model can be summarized into the following five points:

1. The Adversary can eavesdrop, block, and intercept any message on the network.
2. The Adversary can send and re-send messages.
3. The Adversary can combine and decompose messages.
4. The Adversary is familiar with encryption, decryption, hash, and other cryptographic operations. They can perform any encryption operations specified in the protocol, and decrypt encrypted messages when the decryption key is known.
5. The Adversary is a legitimate member of the system. They have been registered with the system and possess all legitimate security parameters.

However, the DY model is no longer sufficient to fully describe the full capabilities of modern adversaries [40]. Therefore, we extend the DY model (to eDY), assuming that an Adversary satisfying the DY model can also have one of the following capabilities:

**eDY1:** When verifying the defense against attacks, such as leaking temporary session information, the Adversary can obtain temporary random numbers to generate session keys.
**eDY2:** When verifying characteristics such as perfect forward secrecy, an Adversary can obtain another entity’s current session key or the long-term key.
**eDY3:** When verifying an attack caused by disclosing a pre-shared secret, an Adversary can obtain the value of a pre-shared secret between other entities.

Note that eDY1-eDY3 are separately attached. Each time, the Adversary is allowed to satisfy only one of eDY1-eDY3, instead of all of them, while verifying a specific attribute. Otherwise, it is considered a Trivial Attack.

### 4.2. Formal Modeling

The formal security protocols verification method based on CPN Tools used in this paper is an incremental attribute verification method based on the state space. It belongs to the category of formal attribute verification [27].

The goal of modeling was to depict the DY Adversary and its extensions in the model based on CPN Tools as accurately as possible, in order to verify the proposed protocol. The proposed CPN security formal model checking method is summarized in the following.

According to the capability of the DY model, it gives the Adversary a vast knowledge base and the ability to split and combine protocol messages, randomly combining all possible messages in each step, in turn forming the state space of each protocol step. The state space is checked to find the states that are responded to by the honest entity. The traces of these states are extracted to judge whether the message accepted by the honest entity is illegitimate. If the message is illegitimate, the protocol has a defect in this step. If not, the protocol is safe for this step, and the testing of subsequent steps can continue.

#### 4.2.1. Modeling of the Proposed Protocol

**Figure 4:**
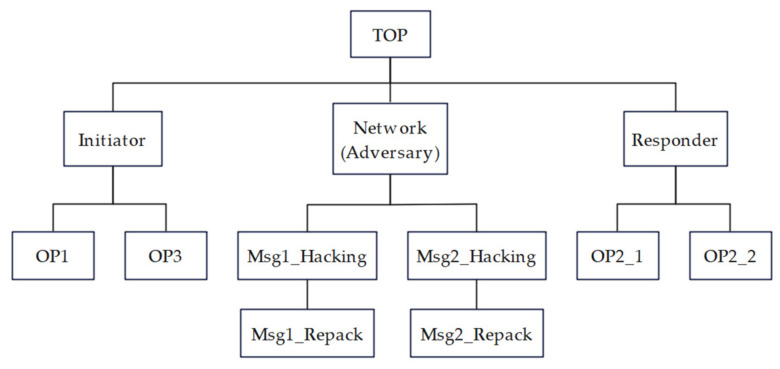
Hierarchical block diagram for a complete view of the model.

In order to keep the CPN model page clear and easy to understand, we use a top-down hierarchical approach to process the model (the CPN model file of this paper can be found in Supplementary Materials). The protocol CPN model consists of 12 sub-pages in 4 layers. Its hierarchical block diagram is shown in Figure 4.

**Figure 5:**
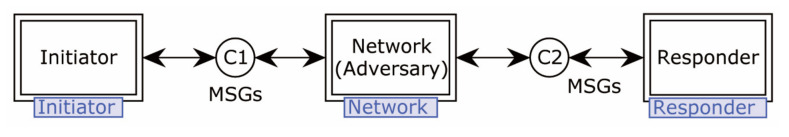
Protocol top-level CPN model.

The top-level CPN model of the protocol is shown in Figure 5.

The initiator and responder communicate through the network interfaces C1 and C2. The adversary controls the network, and the network transition is the adversary.

**Figure 6:**
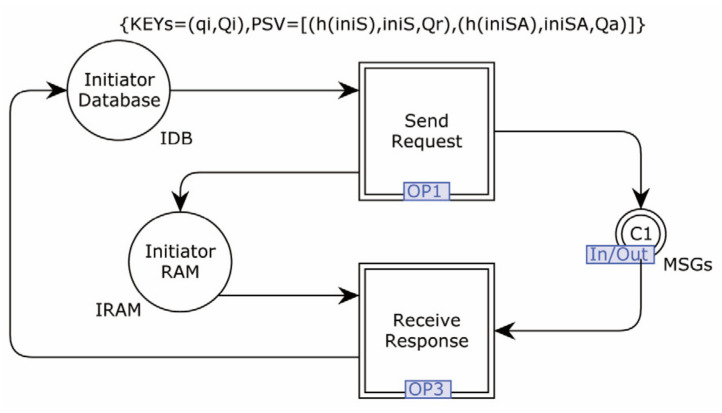
The second-layer model of Initiator.

The second-layer model of the Initiator is shown in Figure 6.

The Initiator’s processing is divided into two sub-pages: OP1 for processing when the request information is sent, and OP3 for processing after the response message is received. The Initiator’s key pair and the pre-shared secret information are stored in the database; in practice, the key pair may be stored in the secure memory, which is only symbolized in the model.

**Figure 7:**
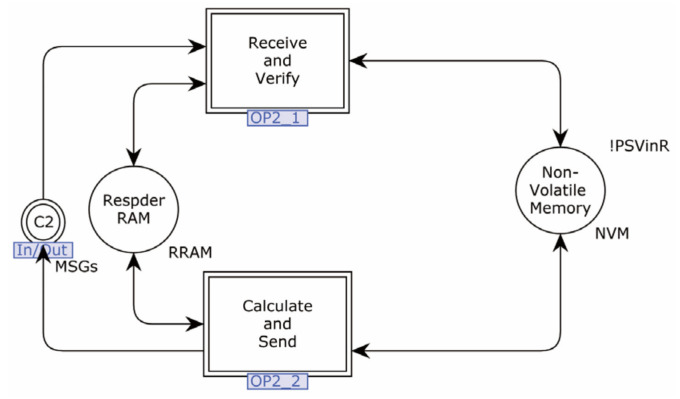
The second-layer model of Responder.

The second-layer model of the Responder is shown in Figure 7.

In this model, the Responder’s processing is divided into two sub-pages. The role of OP2_1 is to process and verify the received request, while the role of OP2_2 is to prepare and send a response message after the message is validated. The non-Volatile_Memory stores the pre-shared secrets of all the devices communicating with it. In addition, the Respder_RAM stores static variables specified by the protocol and temporary data loaded at run-time.

**Figure 8:**
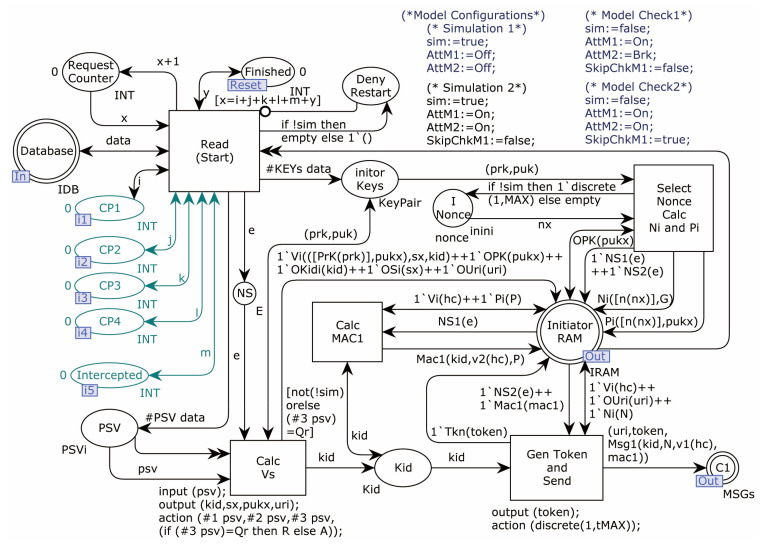
Initiator model sub-page OP1.

The Initiator model sub-page OP1 is shown in Figure 8.

OP1 is the starting page for all model simulation runs, where the Initiator computes and sends the key agreement request. Starting with the transition Read_(Start), the transitions Calc_Vs (calculate the values of v1,v2,v3,v4), Select_Nonce_Calc_Ni_and_Pi (select random numbers and calculate fresh Ni and Pi values), Calc_MAC1 (calculate MAC1), and Gen_Token_and_Send (sends a message) are triggered in turn. According to the DY Adversary model, as the Adversary is also a legitimate member of the protocol, the target objects of Initiator in the model include the legitimate Responder and the considered legitimate Adversary. The sub-page randomly generates two kinds of request messages, targeting the Responder or Adversary. The model temporarily stores the required data in the interface Initiator_RAM for usage by OP3 child pages. In addition, the fusion places CP1–CP4 and Intercepted observe the simulation run result sets, simulating the behavior of the error counter. The places Request_Counter, Finished, and Deny_Restart above are flow control places. The value in Finished keeps track of the number of simulation runs.

**Figure 9:**
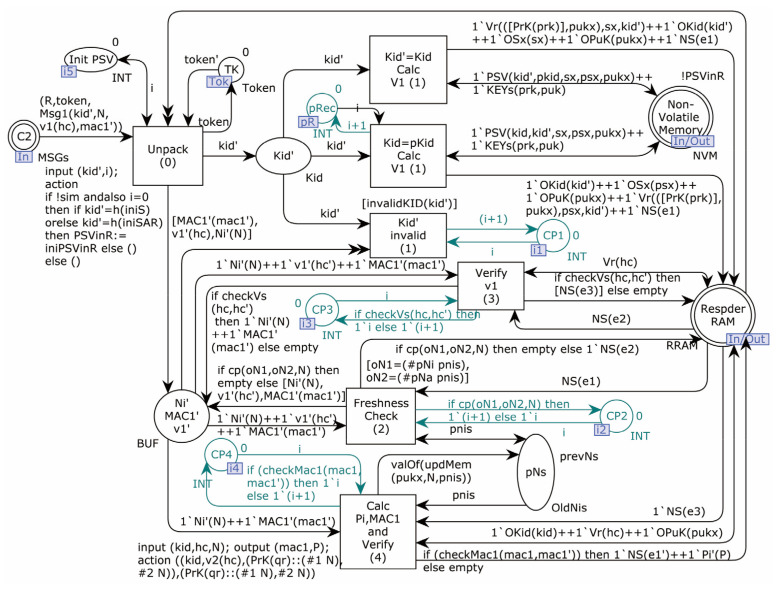
Responder model sub-page OP2_1.

The Responder model sub-page OP2_1 is shown in Figure 9.

Message 1 sent by the Initiator should be received by the Responder OP2_1 sub-page for validation. According to the rules of the protocol, the received Kid’ can be divided into three cases, which trigger one of the transitions Kid’ = Kid_Calc_V1(1), Kid = pKid_Calc_V1(1), or Kid’_invalid(1). If Kid’ does not exist in the store, The transition “Kid’_invalid(1)” triggers, increasing the counter’s value CP1 and terminating the run. If Kid’ exists and is equal to the stored Kid value, the transition Kid’ = Kid_Calc_V1(1) triggers, loading Kid and S into memory. If Kid’ exists and is equal to the stored pKid value, the transition Kid = pKid_Calc_V1(1) is triggered, which loads pKid, and pS into memory as the Kid and S values used in the subsequent calculation. The transition Freshness_Check_(2) is then triggered, in order to determine whether the accepted value of N is not equal to the last stored value of N (pN). If so, the message is discarded, the counter CP2 is incremented by 1, and the operation is terminated. If not, the transition Verify_v1_(3) is triggered, in order to determine whether the received value of v1 equals the calculated value of v1. If not, the process is terminated, and the counter CP3 is incremented by 1. If equal, the transition Calc_Pi, MAC1_and_Verify_(4) is triggered to put the values used to calculate Pi and MAC1 into memory and compare them with the received MAC1’. If not equal, the process terminates, and the counter CP4 is incremented by 1. If equal, it goes to the following sub-page (OP2_2).

**Figure 10:**
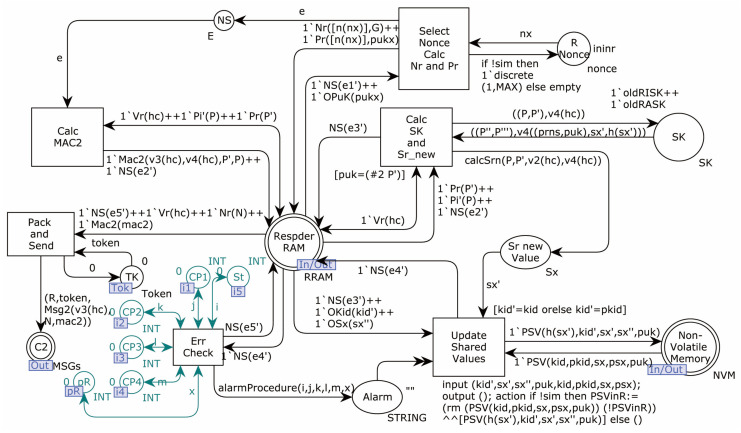
Responder model sub-page OP2_2.

The Responder model sub-page OP2_2 is shown in Figure 10.

The place Alarm in this sub-page is used to display alerts that the model sets in two ways: One is that the fusion CP1–CP4 detects the interrupt caused by the failure of judgment in Responder OP2_1. If this happens, Alarm will get the token “Warning: Attack detection (1)”. The other is caused by the originator sending a message of old shared values, due to a Network outage or desync attack. Alarm will get the token “Notice: Network anomaly (1)” if this happens. During the model simulation run, the number after the token’s prompt message is variable, representing the number of times the anomaly has been found.

**Figure 11:**
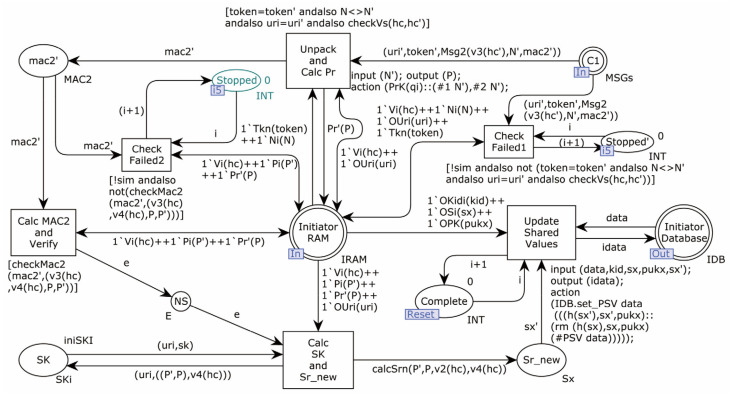
Initiator model sub-page OP 3.

The Initiator model sub-page OP 3 is shown in Figure 11.

This model simulates the process of the Initiator after receiving the response message. In turn, the transition Unpack_and_Calc_Pr is triggered when the received value of v3’ is equal to the value of v3 calculated in the first step, and the received value of N is not equal to the value of N issued in the first step. If these two conditions are not met, the transition Check_Failed1 is triggered, the error counter Stopped’ gets the Token (the original Token+1), and it stops running. If the condition is satisfied, the Pr value is calculated. The transition Calc_MAC2_and_Verify is used to compute MAC2 and verify whether it is equal to the received MAC2’. If not, the transition Check_Failed2 is triggered, and the error counter Stopped obtains the token and stops running. If equal, proceed to the next step. The transition Calc_SK_and_Sr_new is used to calculate SK and Sr_new, and the transition Update_Shared_Values is the last transition in the protocol model, which is used to update the shared secrets in the memory. The fusion place Complete on the page records the number of completions.

#### 4.2.2. Modeling of Adversary

A powerful Adversary can cause a variety of abnormal states and even state space explosion of a protocol. Moreover, as the security attributes of the protocol are related to freshness, the protocol should consider at least two runs, making the CPN model’s running process more challenging to control. When modeling a CPN, we store the previous protocol run directly into the Adversary’s initial knowledge base, in the form of the minor units available to the Adversary, in order to simulate that the Adversary has already executed the protocol with each entity before this simulation. Thus, the process is executed only once, but the results of a second run are simulated. As such, the state space size is effectively reduced.

Furthermore, in order to additionally control the state space size, we adopt incremental verification based on the steps specified by the protocol and perform verification in a step-by-step manner. In the first step of protocol verification, a breakpoint was set after the Responder sent the message, and only the randomly synthesized message of the Adversary and the response message sent by the Responder were analyzed, in order to test the Responder’s ability to resist illegitimate messages. Then, before verifying the second step, let the Adversary in the first step only combine the standard response to the message. In the second step, the Adversary receives a message like this, then sends a random combination to the Initiator. Finally, the state space size is limited to a more appropriate category.

In order to implement the above control, the extension of the Adversary model, and the simulation of the protocol, we designed some configuration items for the model, as listed in Table 2.

**Figure 12:**
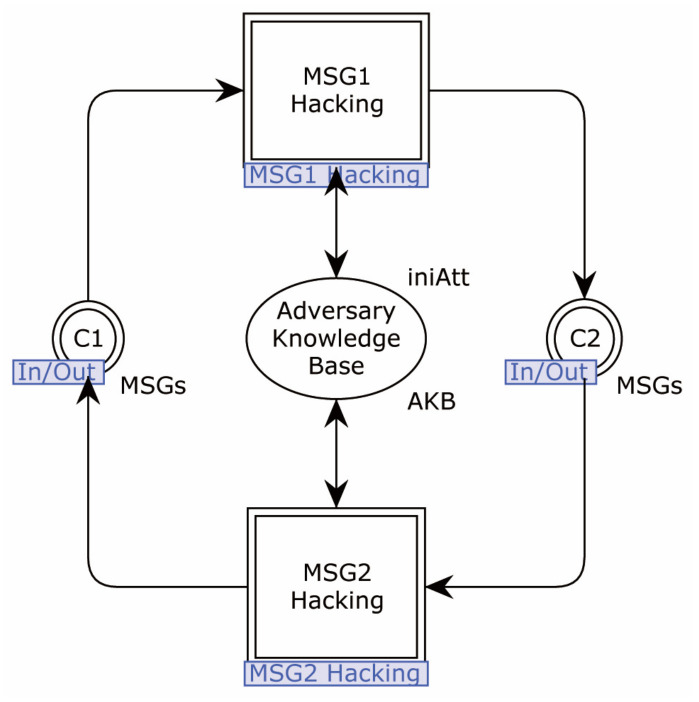
The second-layer page of the network (Adversary).

The second-layer page of the network (Adversary) is shown in Figure 12.

The Adversary is divided into two sub-pages in the model—MSG1_Hacking and MSG2_Hacking—for protocol messages 1 and 2, respectively.

**Figure 13:**
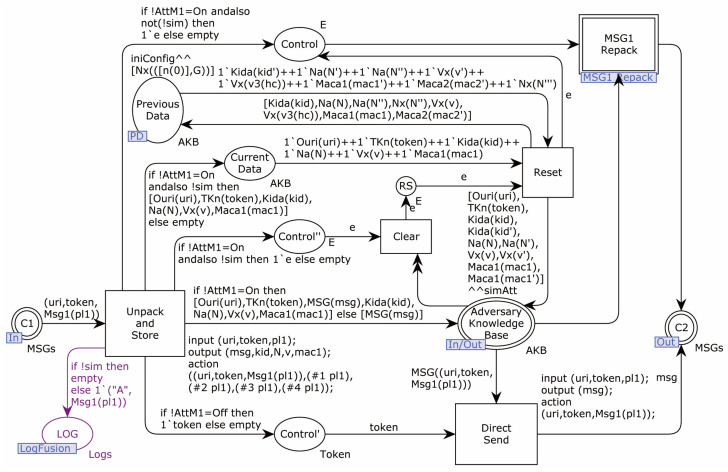
Adversary model sub-page MSG1_Hacking.

The Adversary model sub-page MSG1_Hacking is shown in Figure 13.

After obtaining message 1 sent by the originator, the transition Unpack_and_Store is first triggered to divide the message and store it in the knowledge base. It is then controlled by the configuration item AttM1, in order to decide whether to re-assemble the message. If AttM1 is Off, the transition Direct_Send is triggered to send the message to C2 without processing. Otherwise, the process moves to the MSG1_Repack sub-page. When the configuration item sim is enabled, the transitions Clear and Reset are used to clean up invalid data in the Adversary’s knowledge base, in order to avoid too much data during simulation.

**Figure 14:**
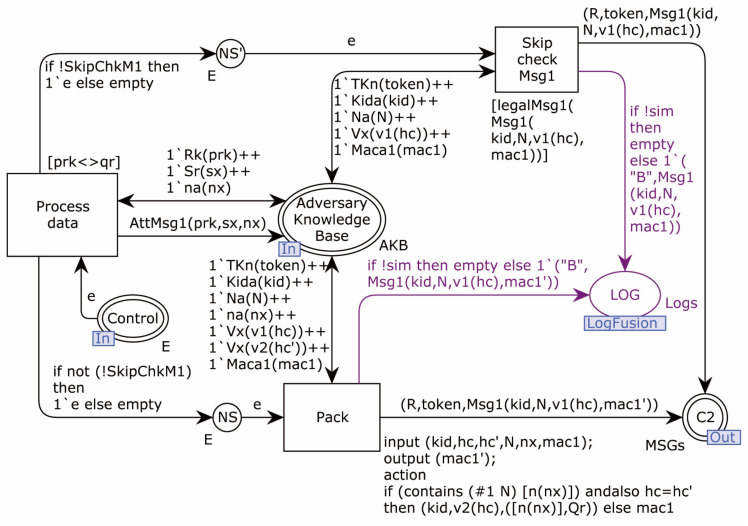
Adversary model sub-page MSG1_Repack.

The Adversary model sub-page MSG1_Repack is shown in Figure 14.

In this sub-page, the Adversary first attempts to compute various possible messages in the format of message 1 using the transition Process_data, based on the data in the existing knowledge base, including legitimate messages communicated to the Adversary. Then, it combines all the new data into the knowledge base. The transition Pack is randomly combined, according to the format of protocol message 1, and the combined message 1 is sent to C2. The role of Skip_check_Msg1 in the page is to skip the random packing by judging the configuration item SkipChkM1 when verifying the Initiator of the second step of the protocol. This transition only combines the request messages that the Responder can respond to, according to the results of the first model checking.

**Figure 15:**
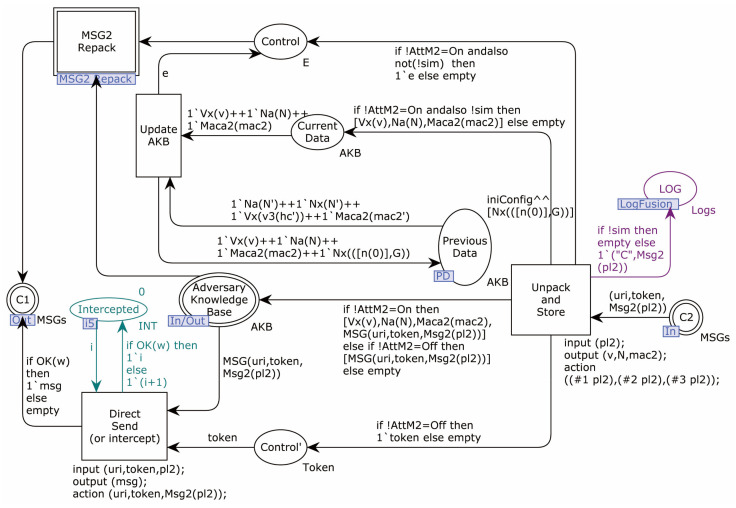
Adversary model sub-page MSG2_Hacking.

The Adversary model sub-page MSG2_Hacking is shown in Figure 15.

As in the Msg1_Hacking sub-page, this sub-page divides the received message 2 into the knowledge base by triggering the transition Unpack_and_Store. According to the value of AttM2, it triggers the transition Direct_Send_(or intercept), or enters the MSG2_Repack sub-page to recombine message 2. The transition Update_AKB is used to update the knowledge base when sim = true and AttM2 = 2, in order to prevent excessive data.

Correspondingly, if the breakpoint AttM2:=Brk is set in the first validation step, it will prevent the transition Unpack_and_Store from firing, and the process will be interrupted there.

Transition Intercepted is for validation to synchronization attack or network failure data loss of counter. When a message loss occurs, its value is increased by one.

**Figure 16:**
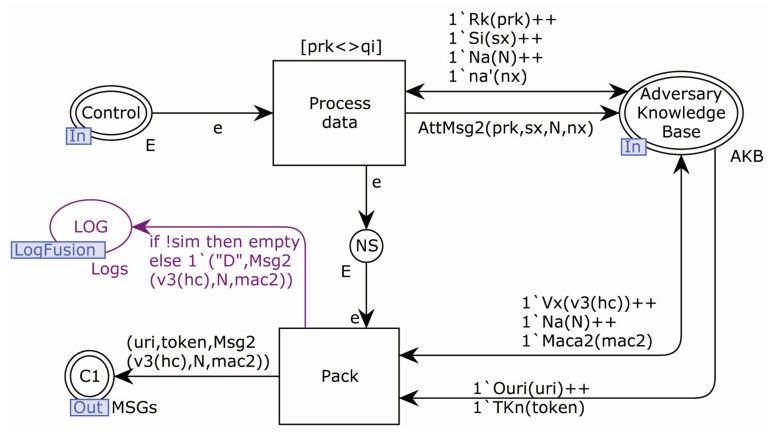
Adversary model sub-page MSG2_Repack.

The Adversary model sub-page MSG2_Repack is shown in Figure 16.

The transition Process_data generates possible messages in message 2 format, puts the generated atomic messages into the knowledge base, and the Pack transition will randomly combine messages, according to the data held in the knowledge base, and send them to C1.

The Adversary’s fusion place LOG stores path messages in an on-the-fly manner. It allows the traces to be extracted quickly after the state space calculation. This place records the contents of the interactive messages for each run in order. The four recorded messages are distinguished by the identifiers “A”, “B”, “C”, and “D”, where “A” represents the request message sent by the Initiator, “B” represents the request message sent to the Responder after tampering by the Adversary, “C” represents the response message sent by the Responder after processing the request message, and “D” represents the response message sent back to the Initiator after tampering by the Adversary.

#### 4.2.3. Security Verification

Due to setting multiple configuration values and observation places in the model, the logic processing becomes more convenient when searching the state space. For example, if the place Complete under the OP3 sub-page in the dead marking has only one Token with the value 1, it means that both the protocol Initiator and Responder have run the protocol entirely once. If there are only three messages in the Adversary’s place LOG of a dead marking, it means that the Responder responds to the message, but the Initiator does not receive it.

According to the eDY model, our validation goal was as follows: Under the premise of excluding the Adversary from delivering and combining legitimate messages, determine whether the honest entity responds to or processes messages randomly combined by the Adversary, and whether the Adversary has all the operands that compute one or more of the secrets of the honest parties after the protocol runs, in a step-by-step manner. If, in the whole state space, the honest entity at each step does not respond or process any illegitimate message randomly reassembled by the Adversary, and the honest entity does not reveal any secret to the Adversary, then the protocol can be considered secure under the eDY model.

The proposed protocol consists of three steps with two interactive messages; therefore, we divided the verification into two cases.

**Case 1:** When verifying the response of the honest Responder in the first step, the configuration items are as follows:

sim:=false; AttM1:=On; AttM2:=Brk; SkipChkM1:=false.

The state trace search condition is set as follows: the Adversary has three records in LOG.

**Case 2:** When verifying the processing of the honest Initiator in the second step, the configuration items are as follows:

sim:=false; AttM1:=On; AttM2:=On; SkipChkM1:=true.

The state trace search condition is set as follows: Complete under the OP3 sub-page of the Initiator has a token, with value of 1.

In addition, the parameter n of extraCap(n) in iniAtt was configured, in order to verify the security attributes of several different Adversary initial values described by the eDY model. The specific meanings of different values are described in Table 3.

Based on the above, we performed state space computations for the two cases under each Adversary configuration, obtaining eight sets of state space reports. The final results are given in Table 4 (all state space reports and screenshots of searches can be found in the Supplementary Materials).

As can be seen from the verification results in the Table 4, we built a vast state space describing the Adversary’s ability in the CPN model. The Adversary sent attack messages that exhausted all possible combinations, but all illegitimate messages were detected and rejected by the honest entity. The Adversary did not obtain all valid data, in order to calculate the secrets of others. When the initial knowledge base configuration in the table was 0 and 2, there were three messages responded to by honest entities after de-duplication, and the trace searching result is:

1. A interacts with Responder typically with the shared Sid of the current round.
2. A interacts with Responder typically with the shared Sid of the previous round (the number of alarms is 1).
3. A transmitted the request sent by Initiator to Responder (without modification).

With initial knowledge base configurations 1 and 3, the Adversary in advance, through illegitimate means, knows the current honest entities private key or fresh random number, so they can calculate the other key materials (i.e., Pi and Pr); however, with no other key materials, the Adversary cannot calculate on or before the session key or secret S.

At this point, the security of the proposed protocol was verified under the eDY model. The analysis concerning the security attributes is presented in Section 5 below.

#### 4.2.4. Protocol Simulation

In addition to the above security verification, we executed the model simulation in CPN Tools to verify the robustness of the network’s protocol under the Adversary’s control. In the CoAP architecture, the Initiator and Responder have a one-to-many relationship, and the Initiator has no limited resources and does not accept messages that have not been requested. Therefore, the main experimental target of our test is the Responder.

**Figure 17:**
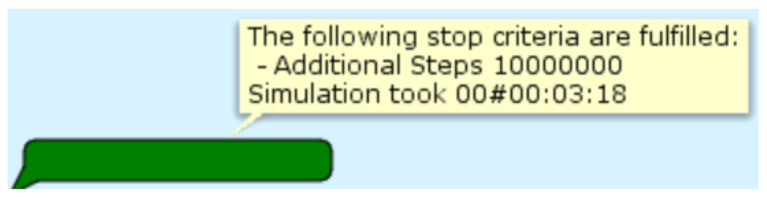
Completion of simulation notice in CPN Tools.

We set the configuration as follows:

sim:=true; AttM1:=On; AttM2:=On; SkipChkM1:=false;

Transitions: 10 million times (see Figure 17).

The purpose of this configuration was to simulate a situation where the Adversary sends numerous deranged messages to the Responder. Furthermore, we observed how the protocol was affected in this case, as well as the outcome of the Responder alert, in order to verify the robustness and survivability of the protocol.

**Figure 18:**
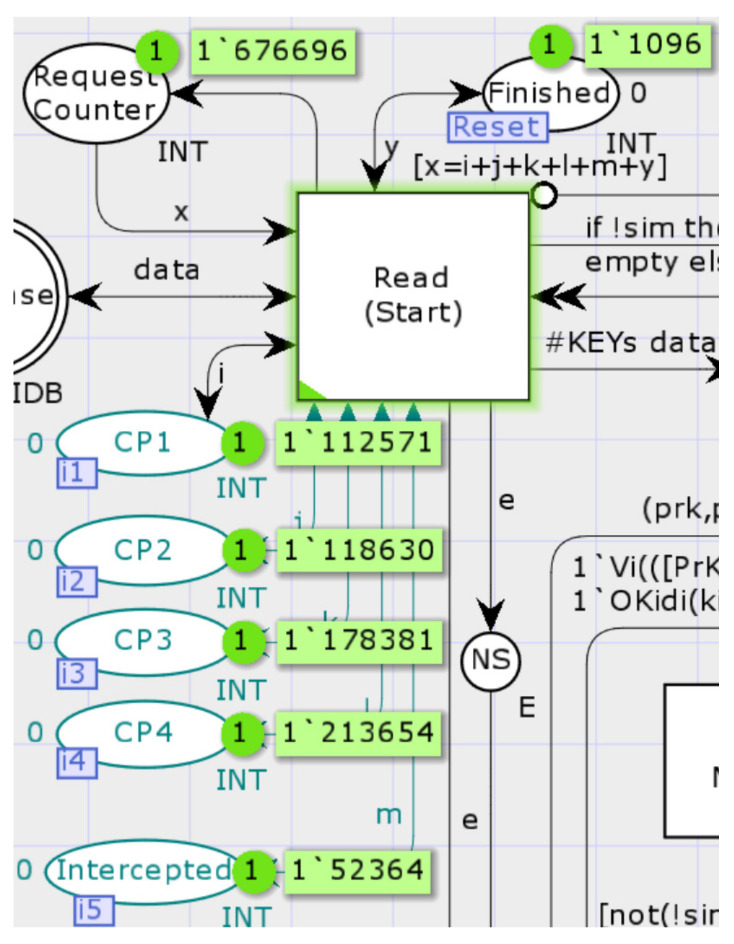
The simulation results in observation places.

When the Adversary controlled the network and re-organized messages at each step of the protocol, the model simulation ran 676,696 times. It can be seen in Figure 18, from the figure, that the simulation results were consistent with the analysis results of the state space above. All attacks against the Responder were detected, and corresponding alerts were sent, except for 1096 cases in which the Adversary happened to combine standard messages from the Initiator and Responder. In the following, the four cases CP1–CP4 are analyzed separately (see Table 5).

The Intercepted token value, which represents the number of interrupts caused by errors found on the Initiator’s side, was 52,364. According to the protocol rules, the Initiator’s shared secret is not updated when it finds an interruption. Thus, this simulates the case of a desynchronization attack.

Consequently, despite numerous attacks and network interruptions, synchronization was preserved between the Initiators and Responders in the protocol. The device sensed and discarded all attack messages, and the interactions between entities could still operate normally after the attack was over. This indicates that the protocol has strong robustness.

It can also be seen, from the experiment, that once the value of the error counter represented by CP1–CP4 changed, it can be concluded that the Adversary had attacked the Responder. The change in Intercepted value indicated an Adversary attack or network failure, providing the system with the possibility of protocol-level attack and anomaly detection, based on which the Responder may send an alert message to the Initiator of the established session when an anomaly is found. Once the network administrator notices the alert message, it can determine whether the network has been compromised at the protocol level.

## 5. Security Analysis

In this section, the proposed protocol’s security attributes and attack resistance are discussed informally, based on the model checking and experimental results presented above.

### 5.1. Confidentiality

The session key and shared secret $S_{r}$ are the data that the proposed protocol must keep secret. According to the state space verification results, the eDY Adversary exhausted all possibilities and failed to obtain any secret between honest entities during the protocol’s operation, indicating that the protocol is confidential. In the two interaction messages, $S_{r}$, $P_{i}$, $P_{r}$, v2, v4, and SK are not transmitted in clear text. ${\mathrm{Kid}}_{r}$ is calculated by hashing a different secret, $S_{r}$, each time. The Adversary cannot reverse hash and, therefore, cannot obtain $S_{r}$. Similarly, as they do not possess the private keys of both parties, they cannot calculate the session key materials $P_{i}$ and $P_{r}$ calculated by elliptic curve point multiplication of fresh random numbers every time and, so, the Adversary cannot obtain SK.

### 5.2. Data Integrity

In the proposed protocol, both sides send messages only once in one interaction, and the receiver can detect abnormal messages. The use of v1, v3, MAC1, and MAC2 ensures data integrity. In message 1, if the Adversary forges ${\mathrm{Kid}}_{r}$, $S_{r}$, or the private key, v1 will fail to verify. If $N_{i}$ is changed, $\mathrm{MAC}1=h\left({\mathrm{Kid}}_{r}{\;||\;v2\;||\;P}_{i}\right)$ will fail to verify. However, in message 2, if v3 is modified, the receiver will first detect and terminate the protocol. If $N_{r}$ is modified, $\mathrm{MAC}2=h\left({v3\;||\;v4\;||\;P}_{r}{\;||\;P}_{i}\right)$ will verify that failure. Therefore, the protocol provides a data integrity guarantee.

### 5.3. Mutual Authentication

MAC1 in message 1 includes v2, data that does not appear in the sent message and cannot be forged. The Responder confirms the identity of the Initiator by verifying MAC1. Similarly, the Responder’s message 2 contains the v4 that has not been sent, and the Initiator verifies the MAC2 that contains v4 to authenticate the Responder.

### 5.4. Perfect Forward and Backward Secrecy

The session key is generated using a random number and combination of private keys and shared secrets. In the state space validation, this attribute was verified. The honest entity’s private key was added to the Adversary’s knowledge base, but the validation results show that the Adversary could not calculate the current or former SK. The Adversary cannot obtain the secret $S_{r}$ every time it changes, as it is based on random numbers; therefore, they cannot generate the session key. Besides, if the Adversary obtains one or more session keys that were used previously, as the sessions are unrelated, they cannot recover useful key material from it, nor can they calculate previous or future session keys. Hence, the protocol has perfect forward and backward secrecy.

### 5.5. Device Anonymity and Unlinkability

The protocol does not send device identity information in plain-text. The public key indices are obtained through the temporary value ${\mathrm{Kid}}_{r}$, and there is no mapping relationship between the value and the actual ID. Under normal circumstances, after the session, ${\mathrm{Kid}}_{r}$ and other message value components are updated. Adversary cannot use ${\mathrm{Kid}}_{r}$ linked to a specific device or other fields.

### 5.6. Resistance to Impersonation Attack

Each party has a private key and shared secrets in the proposed protocol, and the values of S and ${\mathrm{Kid}}_{r}$ are changed every time. Without knowing all the materials, the Adversary cannot impersonate the Initiator and pass the authentication, and it is also impossible to impersonate the Responder by responding to the Initiator with a legitimate message.

### 5.7. Resistance to Man-in-the-Middle Attack

In the proposed protocol, as the Adversary cannot carry out the impersonation attack, it is impossible to insert a forged message into the session for the honest entity to identify and authenticate. The state space verification in Section 4.2.3 verifies the resistance to Man-in-the-Middle attacks; as such, the protocol is immune to Man-in-the-Middle attacks.

### 5.8. Resistance to the Privileged Insider Attack

When modeling in Section 5, we assumed that the Adversary was also a legitimate protocol member and could communicate with honest entities normally. Therefore, this happens to verify the resistance of the protocol to internal privilege attacks in the later state space analysis. In addition, we put the secrets of various other entities into the Adversary’s knowledge base separately. The verification results indicated that the Adversary could not acquire any useful key material, demonstrating that the protocol is resistant to internal privilege attacks.

### 5.9. Resistance to the Known Session-Specific Temporary Information Attack

In this attack, it is assumed that the Adversary has access to temporary random numbers generated by both communicating parties, as such random numbers are usually not in protected memory, and can be exploited by the Adversary if they are not properly removed after the session is executed. Nevertheless, in Section 4.2.3, the state space model validation (case 3) put all the fresh random nonces of honest entities into the Adversary’s knowledge base. It can be seen, from the results, that the Adversary could calculate $P_{i}$ and $P_{r}$, but could not get either side’s private key and, so, could not calculate the key material v4. Therefore, the protocol is also resistant to this attack.

### 5.10. Resistance to the Replay Attack

The Adversary uses the intercepted message to perform a replay attack, in order to convince the communicating party that the Adversary is the honest entity that sent the message. The replay message of the proposed protocol has two types: one involves replaying the previous request by the Initiator, and the other involves replaying the request before the previous one by the Initiator. In Section 4.2.4, the simulation results show that, for the former, as the protocol stores the $N_{i}$ received by the Responder in the previous response, the Responder can determine that the message was repeated, then discard it without processing. For the latter, as ${\mathrm{Kid}}_{r}$ has expired in the message at this time, the Responder finds it in the first step of checking after receiving the message, and the message is also discarded. However, at the Initiator, after receiving the replay message, it verifies v3’ and MAC2 to find and reject the replay attack.

### 5.11. Resistance to the Key Compromise Impersonation Attack

In this case, the Adversary has obtained the long-term key of an honest entity, and attempts to communicate with other devices by pretending to be this entity. In the proposed protocol, this is impossible. All devices, in advance, calculate the Shared secret $S_{r}$. The Adversary, having the private key of everyone else, cannot calculate ${h(q}_{r}Q_{i}{\;||\;S}_{r}{\;||\mathrm{Kid}}_{r})=(v1,\;v2,\;v3,\;v4)$, and cannot be certified by other devices. Therefore, an Adversary cannot carry out a KCI Attack on the proposed protocol.

### 5.12. Resistance to the Desynchronization Attack

The Adversary may intercept the response sent back by the Responder while the protocol is running, such that the Responder updates its storage, believing that it has received a legitimate message, while the Initiator does not receive any response and does not update the stored value. In this way, the Adversary intends to cause a desynchronization attack, such that legitimate parties cannot communicate normally. In Section 4.2.4, our Adversary performed more than 50,000 such attacks in the simulation. However, the honest entities Initiator and Responder were still synchronized, and the shared values used for computation were not inconsistent. This was because the Responder records the previous ${\mathrm{Kid}}_{r}$ and $S_{r}$, and the value stored by the Initiator to calculate the subsequent request is still valid whether or not they receive the response message during the protocol interaction. Therefore, the protocol is not vulnerable to desynchronization attacks.

### 5.13. Key Misbinding Problem

In some schemes based on the elliptic curve (see, e.g., [20,24]), assuming that the party ID is strictly public key bindings, and assuming that the public keys were obtained by the parties in the existing security key distribution, the parties know the public key corresponding to the primary body status. In reality, however, this assumption is impractical. The Adversary can use the public key distribution process, binding the wrong public key to each device ID. In our scheme, the binding is conducted in the form of a shared key index, and the Adversary cannot tamper with the index value and the public key value. Therefore, in our scheme, the binding is in the form of a shared key index and not public binding, and does not need the above assumption.

### 5.14. Resistance to the DoS Attack

The Adversary may send numerous repeated messages to exhaust the device’s resources. According to the simulation experiment results, all attack messages were detected, and CP1–CP4 were directly associated with the error counters. If a large number of error messages are received in a short period, the protocol will detect the intent of a DoS attack, triggering the alarm and subsequent emergency action. In the implementation, the network administrator can set the interval between two authentication requests and, if the value of the error counter increases too fast within a certain period, then the receiving port is blocked for a while, along with other mechanisms to prevent the impact of DoS attacks. In addition, the Responder alarm function may be transferred out-of-band, such that the managers can be found at the beginning of the attack, and the network attack behavior and involvement in the active defense measures can be determined.

### 5.15. Discussion of the Amplification Attack

An amplification attack is a kind of DoS attack. CoAP supports the observe mode and multicast and, so, the Adversary can send a small number of commands to trigger a large number of response data, or even a steady stream of response data, causing network paralysis. Therefore, the proposed protocol is immune to amplification attacks, as the Adversary cannot successfully implement forgery or replay attacks. However, after the session is established, the defense of encrypted message replay and forgery attacks depends on the underlying security protocols. In addition, at the network level, in order to prevent network paralysis, setting allowlists for devices, firewalls, and speed limiting based on IP and port numbers are effective methods to defend against DoS attacks.

## 6. Performance and Security Evaluation

### 6.1. Computation and Communication Overhead Evaluation

We compared the proposed protocol with similar protocols [20,21,22,23,24]. It was assumed that the ID, random number, hash value, point of the elliptic curve (ECC), secret or key, and timestamp (TS) length were 64, 128, 128, 320, 128, and 32 bits, respectively. Furthermore, the public key certificate was assumed to be a C509 certificate (CAu), encoded by COSE [41], with a length of 139 Bytes. The ciphertext (Cip) after symmetric encryption was uniformly calculated using 160 bits, as described in [20] (actually, the ciphertext size of AES is calculated by the plain-text length). In addition, the time consumption of hash calculation ($T_{h}$), elliptic curve point multiplication ($T_{\mathrm{pm}}$), point addition ($T_{\mathrm{pa}}$), symmetric encryption and decryption operation ($T_{e/d}^{S}$), and time stamp operations were 0.00032, 0.0171, 0.0044, and 0.0056 s respectively [23]. The time consumption of other operations was negligible and, thus, ignored.

Therefore, the proposed protocol’s computation overhead is 0.07032 s for each party, and the total is 0.14064 s. The communication overhead is 832 bits for both parties. Compared with those of other protocols proposed in the literature, as detailed in Table 6 and Table 7, as can be seen from the table, the proposed scheme was not the least expensive, but it provided more security and anti-attack attributes than the other schemes, as shown in Table 8. Therefore, we believe that, although the Internet of Things is a restricted environment, the protocol design should be as lightweight as possible without compromising any common security attributes, which involves a trade-off: a protocol may be too lightweight to provide robust security, but we wish not to pay too much computing power for more robust security.

**Table 6:** Comparison of computation overhead with similar schemes.

Protocol	Initiator	Responder	Total
Suman et al. [20]	${4T}_{h}{+3T}_{\mathrm{pm}}{+4T}_{e/d}^{S}$	${3T}_{h}{+3T}_{\mathrm{pm}}{+4T}_{e/d}^{S}$	${7T}_{h}{+6T}_{\mathrm{pm}}{+8T}_{e/d}^{S}$
Abosata et al. [21]	${2T}_{e/d}^{S}$	${2T}_{e/d}^{S}$	${4T}_{e/d}^{S}$
Oliver et al. [22]	${2T}_{e/d}^{S}$	${2T}_{e/d}^{S}$	${4T}_{e/d}^{S}$
Das et al. [23]	${6T}_{h}{+8T}_{\mathrm{pm}}{+4T}_{\mathrm{pa}}$	${6T}_{h}{+8T}_{\mathrm{pm}}{+5T}_{\mathrm{pa}}$	${12T}_{h}{+16T}_{\mathrm{pm}}{+9T}_{\mathrm{pa}}$
Alzahrani et al. [24]	${4T}_{h}{+3T}_{\mathrm{pm}}{+1T}_{\mathrm{pa}}$	${4T}_{h}{+3T}_{\mathrm{pm}}{+1T}_{\mathrm{pa}}$	${8T}_{h}{+6T}_{\mathrm{pm}}{+2T}_{\mathrm{pa}}$
Our Protocol	${6T}_{h}{+4T}_{\mathrm{pm}}$	${6T}_{h}{+4T}_{\mathrm{pm}}$	${12T}_{h}{+8T}_{\mathrm{pm}}$

**Table 7:** Comparison of Communication overhead with similar schemes.

Protocol	Communication Cost	Flights
Suman et al. [20]	$2\left|\mathrm{ID}\right|+4\left|\mathrm{Cip}\right|+1\left|\mathrm{CAu}\right|+4\left|\mathrm{TS}\right|$	4
Abosata et al. [21]	$2\left|\mathrm{ID}\right|+3\left|\mathrm{nonce}\right|+2\left|\mathrm{Cip}\right|$	4
Oliver et al. [22]	$2\left|\mathrm{Cip}\right|$	2
Das et al. [23]	$2\left|\mathrm{ID}\right|+8\left|\mathrm{ECC}\right|+2\left|\mathrm{hash}\right|+3\left|\mathrm{TS}\right|$	3
Alzahrani et al. [24]	$4\left|\mathrm{ECC}\right|+3\left|\mathrm{hash}\right|+2\left|\mathrm{TS}\right|$	3
Our Protocol	$2\left|\mathrm{ECC}\right|+4\left|\mathrm{hash}\right|$	2

**Table 8:** Security attributes and attack-resistant comparison with similar schemes.

Comparative Point		Scheme					
		Suman et al. [20]	Abosata et al. [21]	Oliver et al. [22]	Das et al. [23]	Alzahrani et al. [24]	Ours
Security attributes	Mutual Authentication	√	√	√	√	√	√
	Perfect forward secrecy	×	×	×	√	×	√
	Device Anonymity	×	√	×	×	×	√
	No-synchronized Clocks	×	√	√	×	√	√
	No-stored all expire nonces	√	×	×	√	×	√
Attack Resistance	Replay	√	×	×	√	√	√
	Man in the Middle	√	×	√	×	√	√
	Key Compromise Impersonation	×	×	√	×	×	√
	Privilege-Insider	√	×	√	√	×	√
	Denial of service	×	×	×	√	√	√
	Desynchronization	√	√	×	√	√	√
	Known Specific Temp Info	√	√	×	√	√	√
	Known Key	×	×	×	√	√	√

Note: √ denotes has the attribute; × denotes does not have the attribute.

### 6.2. Evaluation of Security

**Figure 19:**
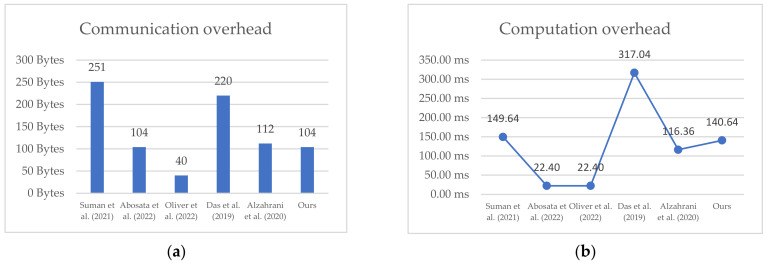
(**a**) Comparison of communication overheads; (**b**) Comparison of computation overheads [20,21,22,23,24].

It can be seen from Figure 19 that our proposed protocol is not the lightest in terms of both Computation and Communication costs among the related works, as it ranks fourth in Computation cost and second in Communication cost; however, our scheme provides more robust security and outstanding features. The following section compares the security and anti-attack abilities of these schemes.

Among the schemes shown in Table 8, the schemes of [21,22] sacrificed part of the security for its extremely lightweight design. Meanwhile, the schemes of [20,23] cannot be applied in a constrained environment due to their high computational and communication overheads caused by the use of public key certificates. Our approach provides a lightweight and high-security solution by combining pre-sharing and public-key cryptography technology, aimed at a mutual compromise between weight and security, and comprises a new solution for authentication and key establishment in M2M environments such as CoAP in the Internet of Things.

## 7. Conclusions

Almost all communication protocols in the Internet of Things face a trade-off between weight and security, and CoAP is no exception. Some of the existing solutions have focused on the security of the protocol, but ignore the weight, while others only consider the computational burden, but pose significant security risks. Among these studies, there have been few privacy considerations. In addition, for historical reasons, the identities of the Initiator and Responder in CoAP implementations are usually reversed, leading to applicability problems for most schemes. In the development of the Internet of Things, vulnerabilities based on security protocols are still a key threat. Exploring the security requirements of various communication protocols for the Internet of Things and providing targeted solutions considering anonymity is bound to improve the security of the Internet of things effectively.

Furthermore, before the advent of quantum computing, there were many reliable cryptographic primitives for protocol designers to choose from, in terms of security verification. Nevertheless, most of the existing security problems arise from the use of these cryptographic primitives, rather than the cryptographic primitives themselves. Consequently, scheme design should not ignore the a priori formal model checking technique. Dolev and Yao were pioneers in this field, contributing the Dolev–Yao Adversary model; however, due to its age, it now has difficulty in accurately depicting the powerful capabilities of modern adversaries. Adversaries can currently infiltrate various parts of the system, and secrets previously thought impossible for adversaries to obtain may now be corrupted. Therefore, improving the Adversary model and its fine-grained formal modeling analysis can significantly improve the possibility of extracting prior vulnerabilities.

In this study, we designed a lightweight security scheme for CoAP of the Internet of Things, which realized anonymous and secure authentication and key agreement functions through the use of standard cryptographic primitives and cooperating with device storage. In terms of verification, we improved the Dolev–Yao Adversary model, assuming that the adversary can obtain the long-term private key, pre-shared secrets, or temporary fresh nonces of the communication parties when verifying different attributes, such that the Adversary has more abilities than that in the original model. The improved model was implemented in a CPN modeling approach, and scheme security analysis was carried out. The results indicate that the proposed protocol provides high security and resistance to most attacks. In addition, compared with other relevant studies in efficiency and security, this scheme is not the most lightweight, but it does provide security attributes and protocol resistance that other schemes do not.

A significant feature of CoAP is its support for multicast, which allows an Initiator to make requests to multiple Responders at the same time. The shortcoming of this work is that we did not consider the multicast feature. Although many other solutions, including DTLS, do not support this paradigm, it is one of the essential features of IoT communication that cannot be ignored.

In future work, we will accumulate more experience in protocol design and consider how to implement group authentication and group key agreement through practical design in the case of multicast, which can improve the efficiency of group authentication while providing increased security. Moreover, formal verification of the Dolev–Yao Adversary model, based on the assumption of perfect cryptography, may fail to verify the defects in the cryptography algorithm. Therefore, exploring how to combine the formal modeling analysis method with a security-proof method, as well as attempting to achieve it within a software environment such as CPN Tools, in order to achieve uncomplicated and automatic verification simultaneously, is considered a worthy direction for our future research.

## Figures and Tables

**Table 1:** Notation and descriptions.

Symbol	Description
${\mathrm{ID}}_{i}{,\;\mathrm{ID}}_{r}$	Identity of Initiator (I), Responder (R)
$n_{i}{,\;n}_{r}$	Random nonce chosen by I and R
p	A large prime number
$F_{p}$	A prime finite field
G	Base point over ${E/F}_{p}$
$Z_{p}^{*}$	A finite field {1, …, p−1}
$\left(q_{i}{,\;Q}_{i}\right),\left(q_{r}{,\;Q}_{r}\right)$	Private and Public key pair of I, R
$S_{r}$ $,\;{\mathrm{pS}}_{r}$	Current and previous secrets shared between I and R.
${\mathrm{Kid}}_{r}{,\;\mathrm{pKid}}_{r}$	Current and previous key index shared between I and R
(v1, …, v4)	The value split by ${h(q}_{i}Q_{r}{||S}_{r}{||\mathrm{Kid}}_{r})$
SK	Session Key between I and R
$N_{i}$ $,\;N_{r}$	The elliptic curve points $N_{i}{=n}_{i}G$ and $N_{r}{=n}_{r}G$
pN	previous $N_{i}$ stored by R
⨁, ||	Bitwise XOR, Concatenation operation
⊥	Null
h(.)	One-way hash function
$E\left(a,b\right){/F}_{p}$	An elliptic curve over prime field $F_{p}$ defined by non-singular elliptic curve equation: $y^{2}{=x}^{3}+\mathrm{ax}+b\;(\mathrm{mod}\;p)$ a, b are constants, $x,y,a,b\;\in {\;F}_{p}$, and $4a^{2}{+27b}^{2}(\mathrm{mod}\;p)\;\neq \;2$

Note: The point multiplication of all elliptic curves in the protocol only retains x coordinates, which will not be described in the following.

**Table 2:** Other initial value configurations.

Variable Name	Type	Position	Effects on the Model
sim	Bool	Pages Pallete	If true, enable loop simulation. Allow setting the transition triggers at specified times.
AttM1	Enum	Pages Pallete	On: enable recombined message 1. Off: message 1 will send without modification.
AttM2	Enum	Pages Pallete	On: enable recombined message 1. Off: message 1 will send without modification. Brk: set a breakpoint where message 2 is received.
SkipChkM1	Bool	Pages Pallete	If true, the Adversary combines only legitimate message 1.
iniAtt	Enum	Pallete	0: Initialize the AKB with the original DY. 1: Initialize the AKB with the original DY and eDY1. 2: Initialize the AKB with the original DY and eDY2. 3: Initialize the AKB with the original DY and eDY3.
LR	Int	Pallete	When sim = true, set the loss rate of message 2.

**Table 3:** Adversary knowledge base initial parameters and verification content.

n	The Initial Knowledge	Verification Content
0	Original data *	Confidentiality, integrity, authentication, Man-in-the-middle Attack, Replay Attack, Device Capture attack, Untraceability, Impersonation Attack
1	Original data and private key of Initiator (I) and Responder (R)	Perfect forward secrecy, Known-Key Attack, Key Compromise Impersonation Attack
2	Original data and pre-shared secret between I and R	Privilege-Insider Attack, Pre-shared Information Disclosure Attack
3	Original data and fresh random nonce of I and R	Known Specific Temporary Information Attack

* The adversary’s initial data according to the original DY model.

**Table 4:** Results of Model Check.

Init KB ^1^	Case	Nodes	Dead Markings	Resp ^2^	Rm Dupl ^3^	Error Count	Illegal Resp ^4^	Key Materials ^5^
0	1	14,304	3322	8	3	1	0	None
	2	29,762	27,004	38	3	1	0	None
1	1	29,868	6972	14	3	1	0	None
	2	85,572	77,408	146	3	1	0	$P_{r}$
2	1	22,086	5147	11	3	1	0	None
	2	54,526	49,506	83	3	1	0	None
3	1	27,004	6280	22	5	2	0	$P_{i}$
	2	101,338	92,108	144	5	2	0	$P_{i}$ $,\;P_{r}$

^1^ The initial Knowledge base of the Adversary. ^2^ The number of nodes that messages were responded to by legitimate entities. ^3^ The number of nodes after removing duplicated response messages. ^4^ The number of nodes that legitimate entities responded to illegitimate messages. ^5^ The Key materials Adversary can obtain when the calculation is done.

**Table 5:** Interpretation of simulation results at CP1–CP4.

Place	Times	Check Value	Adversary Operations
CP1	112,571	Kid_r_	Replayed message 1 eavesdropped before the previous interaction or sent a fake message.
CP2	118,630	N_i_	Replayed previous message 1 directly.
CP3	178,381	v1	Modified the v1 in the message sent by the Initiator.
CP4	213,654	MAC1	Modified Ni in the eavesdropped previous message.

## Data Availability

Not applicable.

## Supplementary Materials

The following supporting information can be downloaded at: https://www.mdpi.com/article/10.3390/s22197191/s1, Folder S1: State space and verification results; File S1: CPNmodel.cpn. Folder S1 contains all state space reports and screenshots of state space searches. File S1 is the complete CPN model file for this article, it can be opened using CPN Tools 4.0.1.
