# Supplementary figures and images for: Lightweight Anonymous Authentication and Key Agreement Protocol Based on CoAP of Internet of Things
Source: Sensors (Basel). 2022 Sep 22;22(19):7191. doi: 10.3390/s22197191 (PMC9573209; doi:10.3390/s22197191)

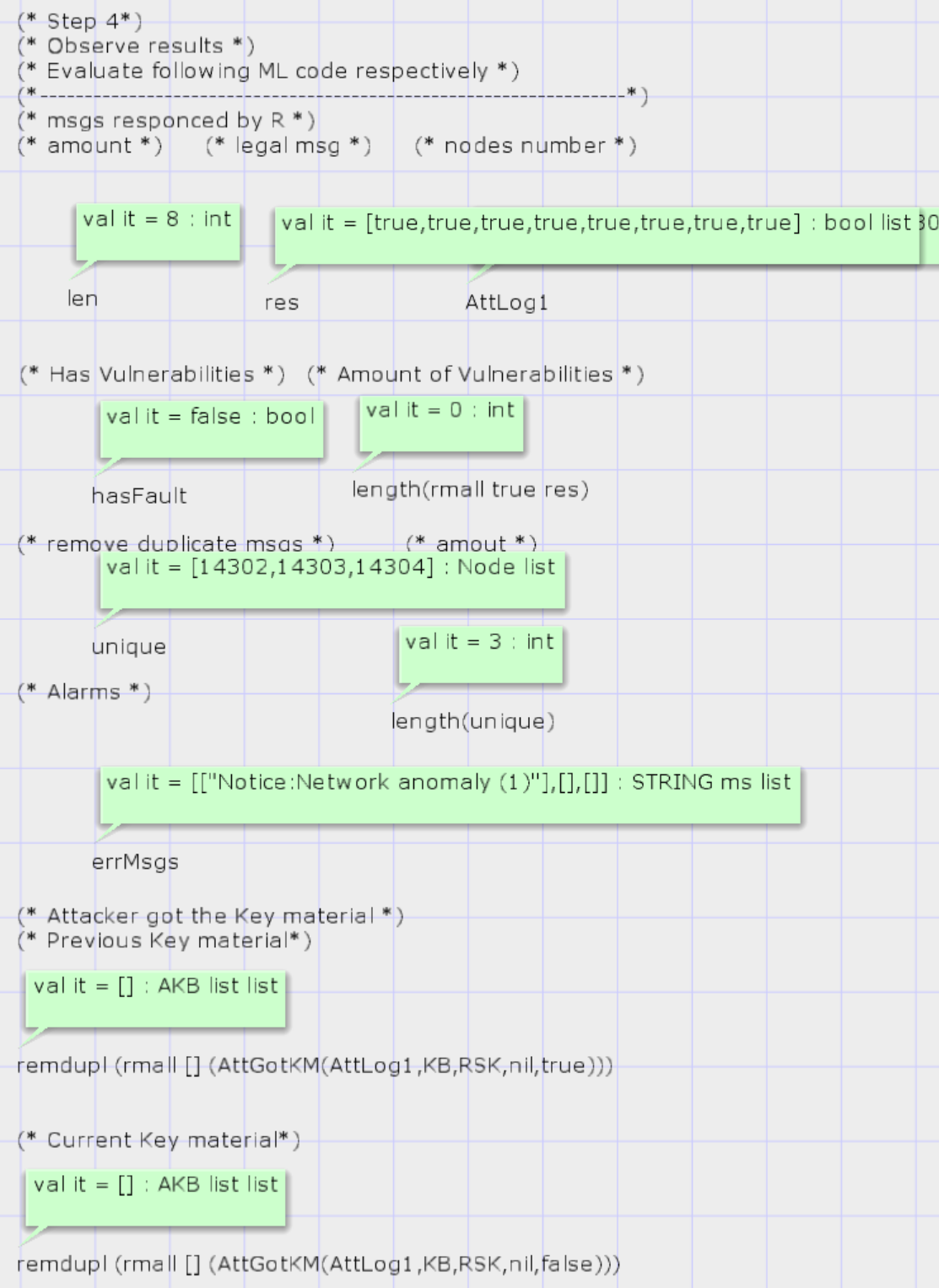

Supplement: Supplementary file 1 [file sensors-22-07191-s001.zip › S1 State space and verification results/0.1.png]

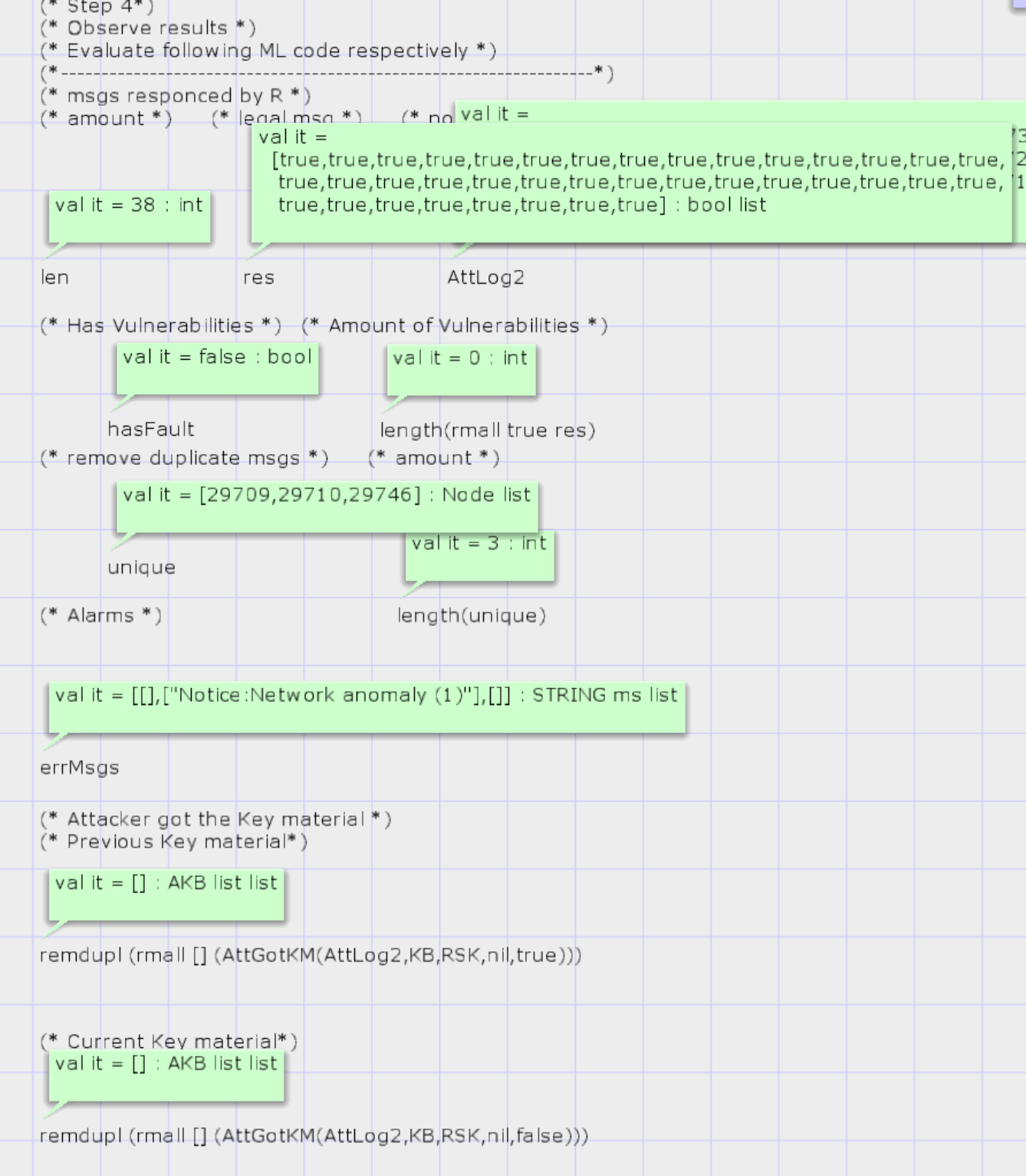

Supplement: Supplementary file 1 [file sensors-22-07191-s001.zip › S1 State space and verification results/0.2.png]

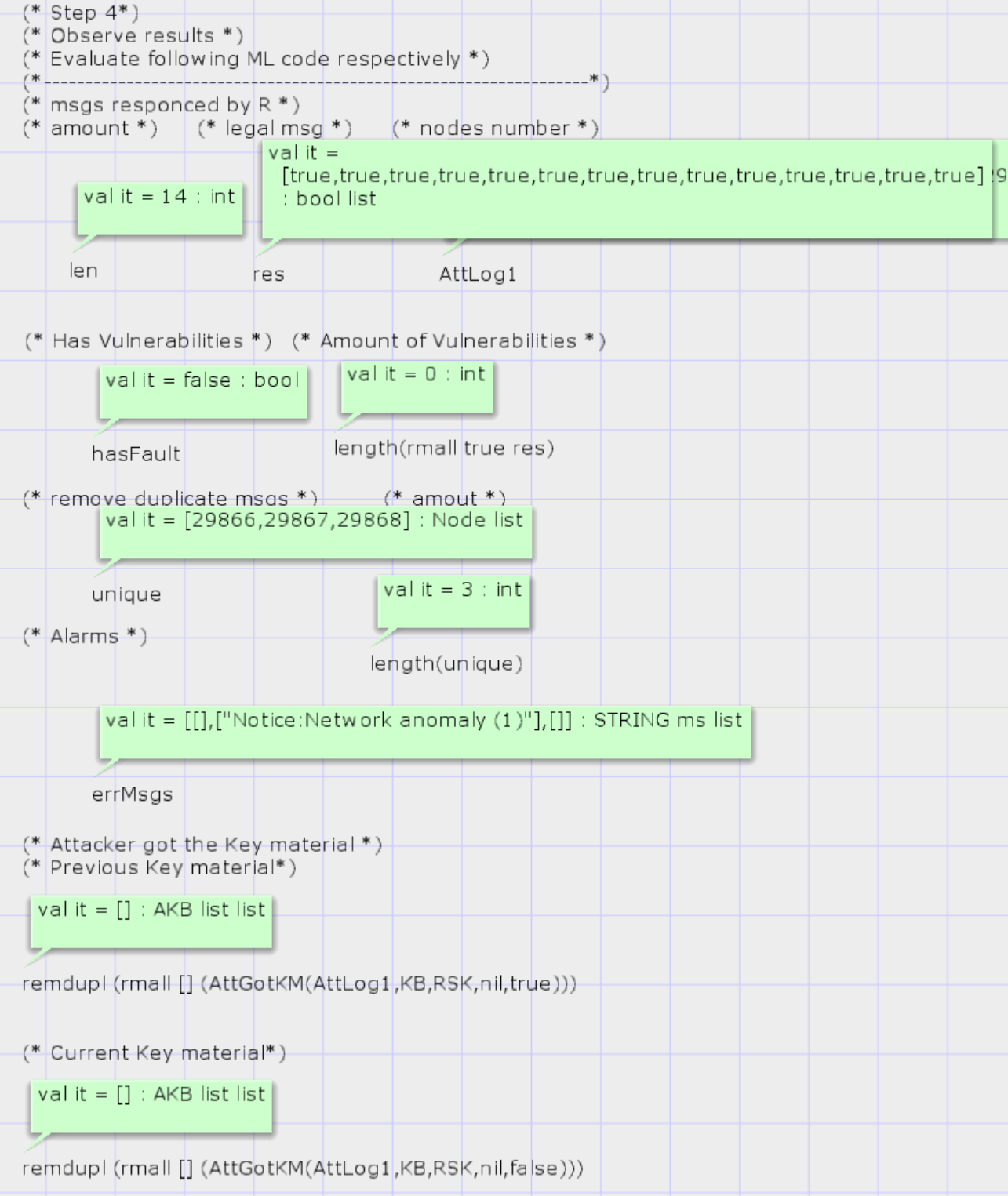

Supplement: Supplementary file 1 [file sensors-22-07191-s001.zip › S1 State space and verification results/1.1.png]

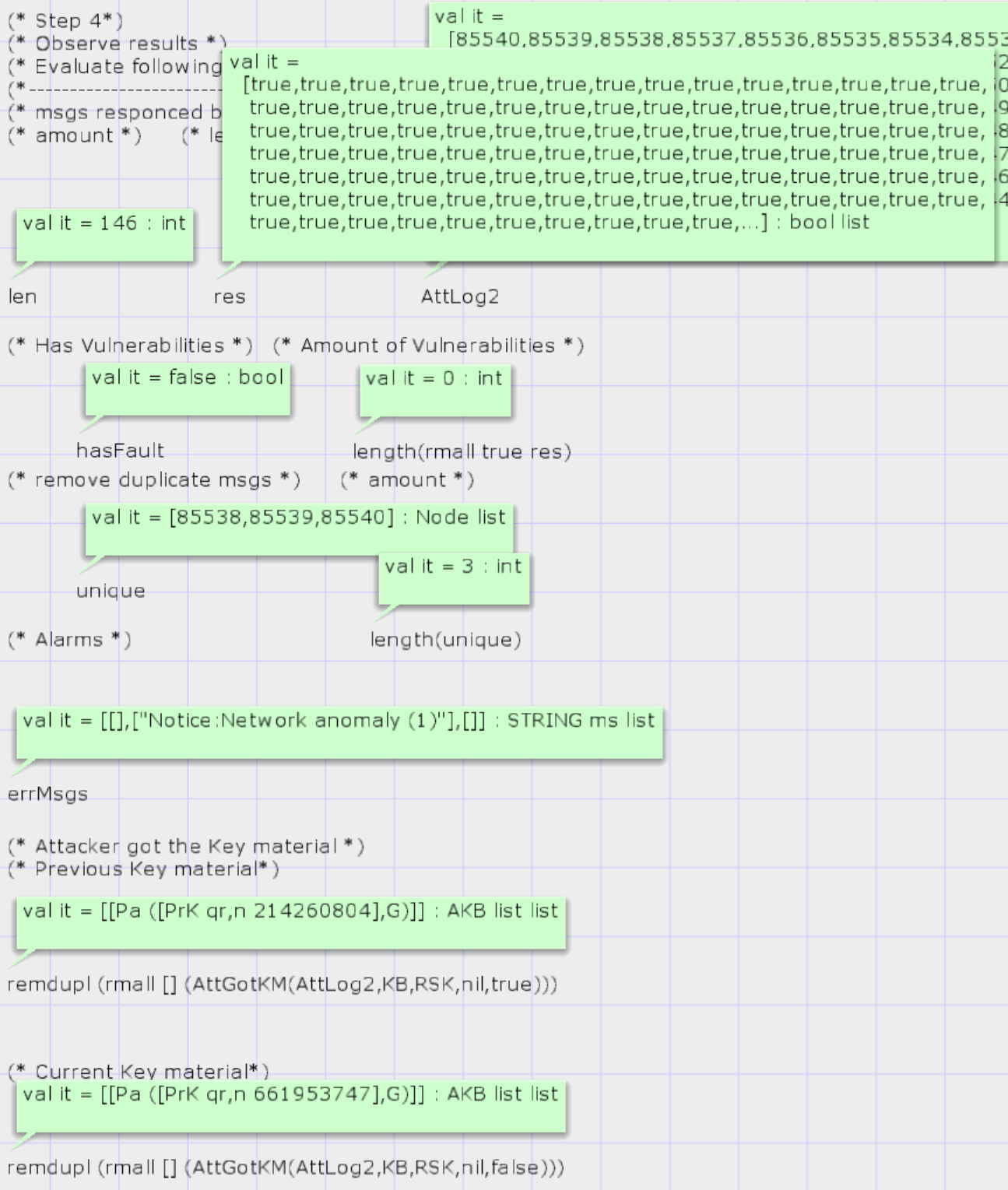

Supplement: Supplementary file 1 [file sensors-22-07191-s001.zip › S1 State space and verification results/1.2.png]

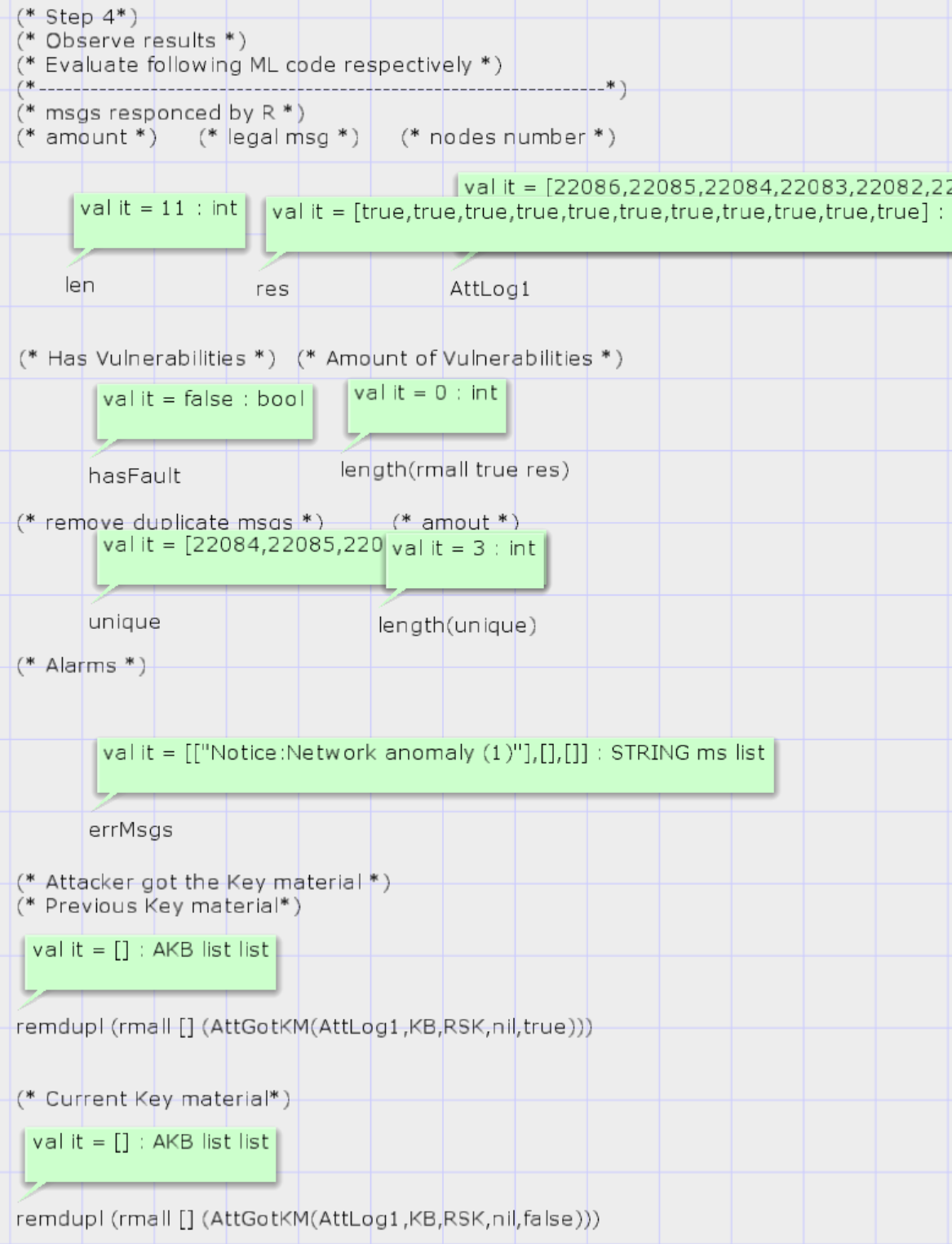

Supplement: Supplementary file 1 [file sensors-22-07191-s001.zip › S1 State space and verification results/2.1.png]

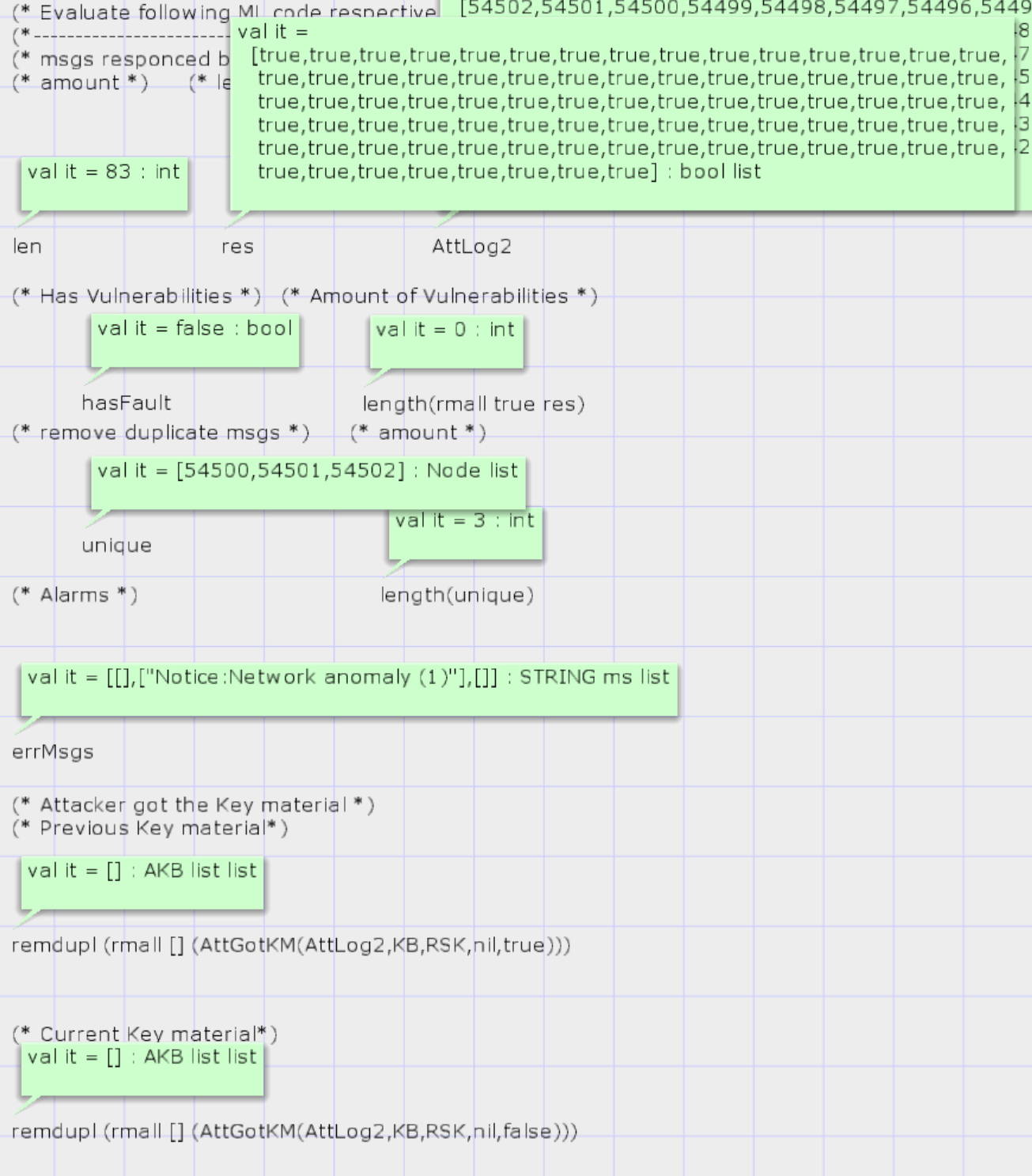

Supplement: Supplementary file 1 [file sensors-22-07191-s001.zip › S1 State space and verification results/2.2.png]

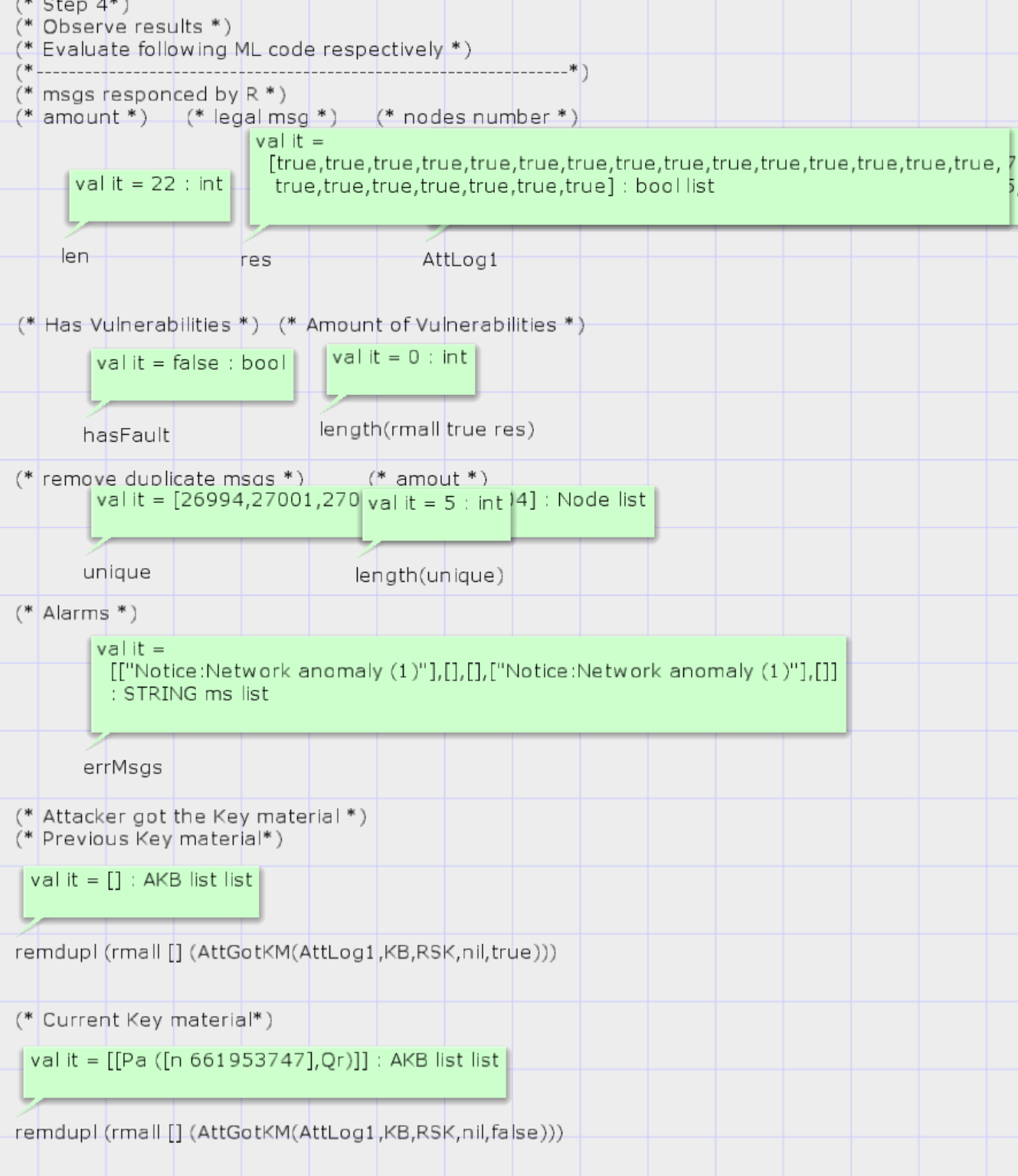

Supplement: Supplementary file 1 [file sensors-22-07191-s001.zip › S1 State space and verification results/3.1.png]

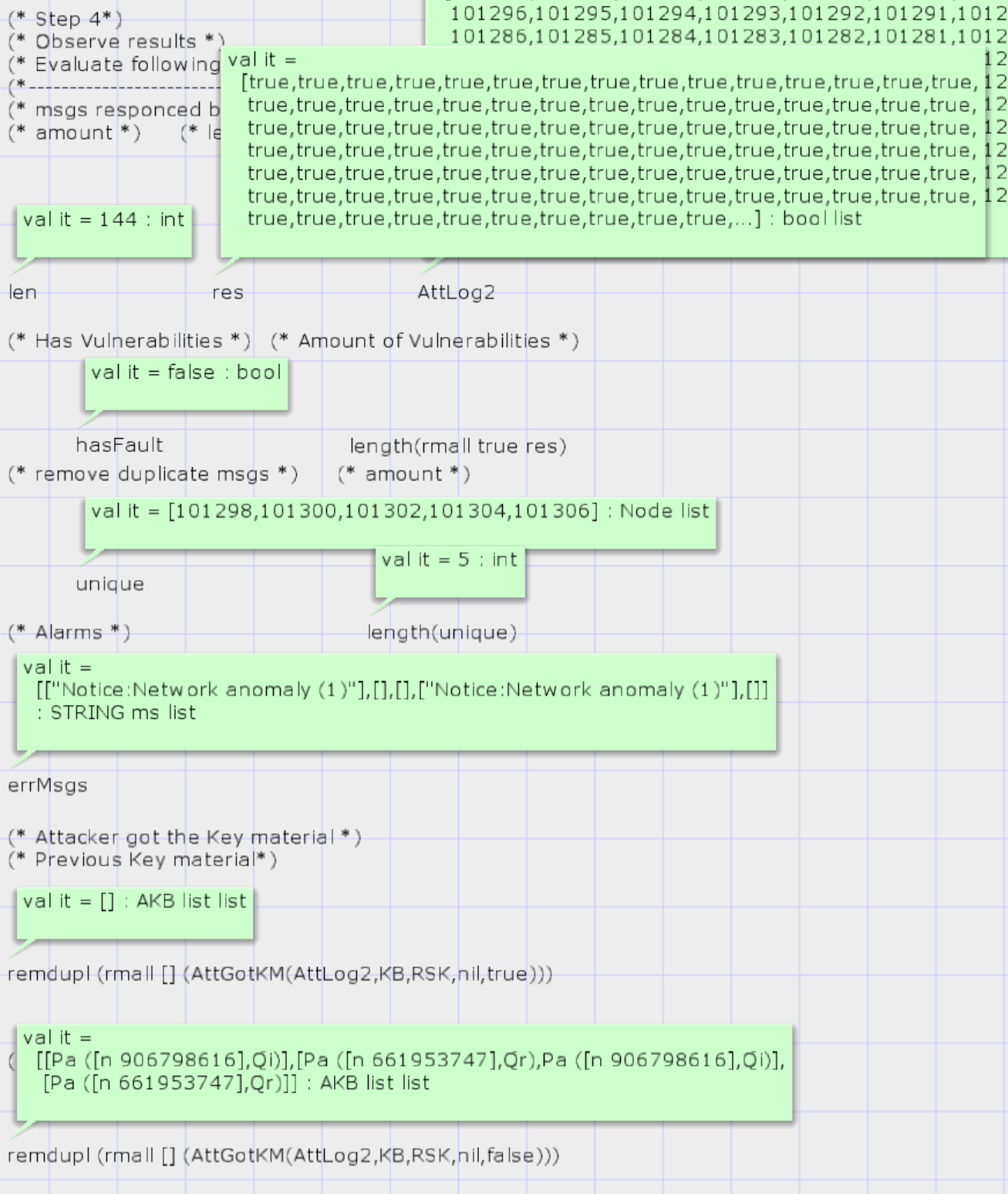

Supplement: Supplementary file 1 [file sensors-22-07191-s001.zip › S1 State space and verification results/3.2.png]
